# Extracellular vesicles derived from oesophageal cancer containing P4HB promote muscle wasting via regulating PHGDH/Bcl‐2/caspase‐3 pathway

**DOI:** 10.1002/jev2.12060

**Published:** 2021-03-10

**Authors:** Xiaohan Gao, Yan Wang, Fang Lu, Xu Chen, Di Yang, Yiren Cao, Weimin Zhang, Jie Chen, Leilei Zheng, Guangchao Wang, Ming Fu, Liying Ma, Yongmei Song, Qimin Zhan

**Affiliations:** ^1^ State Key Laboratory of Molecular Oncology National Cancer Center/National Clinical Research Center for Cancer/Cancer Hospital Chinese Academy of Medical Sciences and Peking Union Medical College Beijing China; ^2^ Key Laboratory of Carcinogenesis and Translational Research (Ministry of Education/Beijing) Laboratory of Molecular Oncology Peking University Cancer Hospital & Institute Beijing China; ^3^ Department of Ophthalmology West China Hospital Sichuan University Chengdu China

**Keywords:** apoptosis, cachexia, oesophageal cancer, extracellular vesicles, inhibitor, muscle wasting

## Abstract

Cachexia, characterized by loss of skeletal muscle mass and function, is estimated to inflict the majority of patients with oesophageal squamous cell carcinoma (ESCC) and associated with their poor prognosis. However, its underlying mechanisms remain elusive. Here, we developed an ESCC‐induced cachexia mouse model using human xenograft ESCC cell lines and found that ESCC‐derived extracellular vesicles (EVs) containing prolyl 4‐hydroxylase subunit beta (P4HB) induced apoptosis of skeletal muscle cells. We further identified that P4HB promoted apoptotic response through activating ubiquitin‐dependent proteolytic pathway and regulated the stability of phosphoglycerate dehydrogenase (PHGDH) and subsequent antiapoptotic protein Bcl‐2. Additionally, we proved that the P4HB inhibitor, CCF642, not only rescued apoptosis of muscle cells in vitro, but also prevented body weight loss and muscle wasting in ESCC‐induced cachexia mouse model. Overall, these findings demonstrate a novel pathway for ESCC‐induced muscle wasting and advocate for the development of P4HB as a potential intervention target for cachexia in patients with ESCC.

## 1 INTRODUCTION

Oesophageal cancer has become the eighth most common malignancy and the sixth leading cause of cancer mortality worldwide (Kamangar et al., 2006). Oesophageal squamous cell carcinoma (ESCC) is the major histopathological subtypes of oesophageal cancer, constituting greater than 80% of oesophageal cancer cases (Chen et al., 2017). The majority of patients with ESCC are accompanied by systemic inflammation, ongoing muscle and weight loss (Diakowska et al., 2010), which is commonly acknowledged as the hallmark characteristic of cachexia (Shyh‐Chang, 2017). Cachexia, which cannot be reversed by conventional nutritional support, represents a fatal energy‐wasting syndrome arising from complex host–tumour interactions in up to 30% of patients with tumours. It is a determinant of tolerance to treatment and survival of cancer patients (Fearon et al., 2011), and the immediate cause of 15% of all cancer deaths worldwide (Klose et al., 2016). Patients with ESCC, similar to those with pancreatic cancer, are more likely to undergo weight loss than patients with other cancer types (Anandavadivelan & Lagergren, 2016). Although muscle degradation triggered by tumour‐derived signalling factors has been reported in prostate cancer, lung cancer and colon cancer (Argilés et al., 2014), the reasons related to ESCC‐induced muscle wasting remain under‐recognized. Thus, it is imperative to decipher the mechanisms underlying the high prevalence of weight loss existed in these patients with ESCC.

Extracellular vesicles (EVs) secreted from different cell types, can be released to the extracellular space via fusion with plasma membrane, and participate in intercellular communication (Théry, 2011). Cargos contained in EVs, such as functional proteins, mRNA and microRNA (miRNA), can be internalized by recipient cells and influence the biological functions of these cells (Melo et al., 2014; Peinado et al., 2012). Previous studies demonstrated that the HSP70/90 and miRNAs in EVs could work as tumour‐derived signalling factors for triggering muscle degradation (He et al., 2014; Zhang et al., 2017), suggesting the pivotal role of EVs in cancer‐associated cachexia. However, whether EVs derived from ESCC can confer certain molecules to trigger cancer‐induced muscle wasting remains to be elucidated.

Prolyl 4‐Hydroxylase Subunit Beta (P4HB), as a member of protein disulfide isomerase (PDI) family, can catalyze thiol‐disulfide exchange and assist in the formation of disulphide bond (Edman et al., 1985; Song & Wang, 1995; Vuori et al., 1992). P4HB engages a cell death cascade when it accumulates at high levels in response to misfolded proteins (Hoffstrom et al., 2010). Our previous whole‐genome and whole‐exome sequencing studies (Cheng et al., 2016; Song et al., 2014) identified an amplification region encompassing P4HB, with high occurrence frequency (9.09%) in 154 ESCC cases. P4HB was reported to be upregulated in patients with gastric cancer and correlated with their poor prognosis (Zhang et al., 2018), and its overexpression could promote hepatocellular carcinoma cell growth and EMT (epithelial‐to‐mesenchymal transition) in vitro (Xia et al., 2017). Furthermore, epigenome‐wide association study revealed the strong association (1.7 × 10^−16^) of P4HB with body mass index (Wahl et al., 2016), and functional study unveiled P4HB mediated mechanical stretch stress and/or advanced glycosylation end products‐triggered proliferation and apoptosis of vascular smooth muscle cells (Ping et al., 2017). However, the role of P4HB in ESCC‐associated cachexia remains unravelled. In the present study, we first developed an ESCC‐induced cachexia mouse model using YES2 cell line, and demonstrated that P4HB was a crucial mediator of muscle wasting in vitro and in vivo. Additionally, treatment with a P4HB inhibitor, CCF642, inhibited myoblast apoptosis in vitro, and ameliorated body weight loss and muscle wasting in vivo, which provides a novel strategy in treatment of ESCC‐associated cachexia.

## 2 MATERIALS AND METHODS

### 2.1 Cell culture

Ten human ESCC cell lines, including YES2, KYSE30, KYSE70, KYSE140, KYSE180, KYSE410, KYSE450, KYSE510, COLO680N, were examined in this study, which were kindly provided by Professor Yutaka Shimada from Kyoto University, Japan. The cells were maintained in RPMI‐1640 (Gibco, Thermo Fisher) culture medium supplemented with 10% foetal bovine serum (FBS). ESCC cell line KYSE150 was cultured in a 1:1 mixture of RPMI 1640 and F12 (Gibco, Thermo Fisher) culture medium with 2% FBS. The immortalized oesophageal epithelial cell lines NE2 and NE3 were provided by Professor Enmin Li from Shantou University, China. These cells were cultured in a 1:1 mixture of EpiLife and dKSFM (Gibco) as described previously (Zhang et al., 2006). 293T, LLC, C2C12 and L6 cells were cultured in DMEM (Gibco) medium containing 10% FBS. Murine skeletal muscle satellite cells were purchased from CHI Scientific, China. The cells were maintained in DMEM/F12 medium supplemented with 10% FBS. Human gastric adenocarcinoma cell line AGS was provided by The Fourth Hospital of Hebei Medical University, China. Two human pancreatic adenocarcinoma cell lines AsPC1 and BxPC3 were provided by Peking Union Medical College Hospital, China. AGS, AsPC1 and BxPC3, were maintained in RPMI‐1640 (Gibco) medium supplemented with 10% FBS. The stable overexpressed cell line, KYSE150‐LV‐Flag‐P4HB, was cultured in 1:1 mixture of RPMI 1640 and F12 (Gibco) with 2% FBS and 0.5 μg/ml puromycin. The stable knockdown cell line, YES2‐shP4HB, was cultured in RPMI 1640 (Gibco) with 10% FBS and 0.5 μg/ml puromycin. The stable knockdown cell line, C2C12‐shP4HB, was cultured in DMEM (Gibco) medium containing 10% FBS and 0.5 μg/ml puromycin.

C2C12 myoblasts were plated in 6‐well or 12‐well plates and cultured in DMEM supplemented with 10% FBS. Upon confluency, the medium was changed to DMEM containing 2% horse serum for 4 days to induce differentiation and the medium was changed every 48 h.

All of these cells were maintained at 37°C with 5% CO_2_.

### 2.2 Animal experiments

All animal care and procedures were in accordance with national and institutional policies for animal health and well‐being and approved by Cancer Institute and Hospital, Chinese Academy of Medical Sciences and Peking Union Medical College (Beijing, China). Male BALB/c nude mice aged at 5 weeks and male C57BL/6J mice aged at 6 weeks were purchased from Vital River Laboratory Animal Technology Co. Ltd (Beijing, China). After a 7‐day acclimation phase, mice were randomized for groups and implanted for xenograft tumour formation.

For generation of ESCC‐associated cachexia model, 3×10^6^ YES2 cells (100 μl) or an equal volume of phosphate buffered saline (PBS) were injected subcutaneously into the right flanks of BALB/c nude mice. Body weight and tumour size (Tumour volume was calculated by the formula: larger diameter × smaller diameter square/2.) were measured twice a week. When body weight reduced over 20%, mice were sacrificed to measure the body weight (Tumour weight was excluded from body weight.), gastrocnemius (GA) muscle weight, tumour weight and foot length (Foot length was measured from heel to tiptoe.). GA muscle was then excised and embedded in paraffin for haematoxylin and eosin staining (H&E) analysis. Quantification of cross‐sectional areas of H&E stained muscle sections was analyzed by the ImageJ software (NIH). For the pair‐fed group, food was weighed every two days. Data from the pair‐fed group were shifted back two days to ensure equivalent food intake for comparisons with tumour‐bearing mice.

For P4HB in xenograft studies, 2×10^6^ KYSE150‐LV‐Flag‐P4HB (100 μl) or KYSE150‐LV‐Control cells (100 μl) were injected subcutaneously into the right flanks of BALB/c nude mice. Other treatments were followed by procedures outlined previously.

For generation of a mouse cachexia model of Lewis lung carcinoma (LLC), 1×10^6^ LLC cells (100 μl) or an equal volume of phosphate buffered saline (PBS) were injected subcutaneously into the right flanks of C57BL/6J mice. Body weight and tumour size (Tumour volume was calculated by the formula: larger diameter × smaller diameter square/2.) were measured three times a week. After 18 days, mice were sacrificed to measure the body weight (Tumour weight was excluded from body weight.), GA weight, and tumour weight. GA muscle was then excised and embedded in paraffin for H&E analysis. Quantification of cross‐sectional areas of H&E stained muscle sections was analyzed by the ImageJ software (NIH).

### 2.3 EV isolation, quantitation and characterization

EVs isolation and identification followed the MISEV2018 guidelines (Thery et al., 2018). Cells were cultured and allowed to reach 80–90% confluence, then replaced with serum starvation. The supernatant was collected after incubation for 48 h and centrifuged at 300 g for 10 min to remove dead cells and cell debris. Then, the supernatant was filtered through a 0.22 μm Millex‐GV filter unit (Millipore) and concentrated to 1 ml using Amicon Ultra‐15 Centrifugal Filter Unit (Millipore, USA). The lipids left in the tube were transferred to a 1.5 ml microcentrifuge tube. EVs were isolated using the ExoQuick‐TC kit (System Biosciences, USA) according to manufacturer's protocol. The precipitation was resuspended in PBS.

EVs were also isolated with ultracentrifugation method to confirm the effects of EVs that isolated with the ExoQuick‐TC kit. Cells were cultured and allowed to reach 80%–90% confluence, then replaced with serum starvation. The supernatant was collected after incubation for 48 h. The culture medium was centrifuged at 300 g for 10 min, 3000 g for 30 min and then 10000 g for 30 min. Then, the supernatant was ultracentrifuged at 110,000 g for 70 min at 4°C. The precipitation was washed with 1 ml PBS and ultracentrifuged at 110,000 g for 70 min at 4°C. The pellets were resuspended in PBS. EV protein quantification was obtained using Pierce BCA Protein Assay Kit (Thermo Fisher Scientific, USA) according to manufacturer's protocol.

EVs were processed by negative staining and observed with transmission electron microscope (TEM‐1400 Plus) at 80 kV. NanoSight NS300 instrument (Malvern Instruments Ltd, UK) was used to examine the size distribution and the concentration of the vesicles, which were analyzed by Nanoparticle Tracking Analysis (NTA) V3.0 analytical software (Malvern Instruments Ltd. UK). EVs were diluted with PBS to 1 ml. The measurement conditions were made at 20.95 ± 0.55°C, 25 frames per second for 60 s with similar detection threshold for each sample. Three recordings were performed for each sample.

### 2.4 Immunohistochemistry

The tissue microarray (HEsoS180Su07) used in this study, including total 103 human ESCC samples and 77 samples of matched adjacent normal tissues, was purchased from Shanghai Outdo Biotech Co. Ltd. The Supplementary Table 2 summarized the detail clinicopathological characteristics.

The immunohistochemistry assay was performed as previously described (Zhang et al., 2017), using anti‐P4HB antibody (1:20000; Proteintech). The scores were determined by combining the intensity of staining and the proportion of positively stained tumour cells as described in the previous paper (Hao et al., 2001). First, the intensity was graded as follows: 0, negative; 1, weak (0, or 1+); 2, moderate (1‐2+, or 2+); 3, strong (2‐3+, or 3+). Second, the proportion of positive tumour cells was graded: 0, <5%; 1, 5%–25%; 2, 26–50%; 3, 51%–75%; 4, >75%. A final score was derived by multiplication of these two primary scores. Final scores of 0–3 were defined as ‘Low expression’; scores of 4–6 as ‘Medium expression’; scores of 7–12 as ‘High expression’. Additionally, for the analysis of association between P4HB expression and clinicopathological features, the final scores of 0–6 were defined as ‘Negative expression’ and scores of 7–12 as ‘Positive expression’.

### 2.5 Plasmid construction and oligonucleotide transfection

The plasmid Flag‐P4HB containing the full‐length coding region of P4HB and fusing with triple flag tags in the C‐terminal, plasmid GST‐PHGDH, V5‐PHGDH were constructed from Shanghai Generay Biotech Co. Ltd. Specific siRNAs targeting P4HB (siP4HB: 5′‐GCTTCAAGGGCAAGATCCTGTTCAT‐3′) were purchased from Invitrogen (USA) and siRNAs targeting PHGDH (siPHGDH: 5′‐GAAAGAGGCTGGCCTCAAT‐3′) were purchased from RiboBio Co. Ltd (Guangzhou, China). Cells were seeded at 6‐well culture plates and transfected with corresponding plasmids or siRNA using lipofectamine 2000 (Life Technologies, Carlsbad, CA, USA) according to manufacturer's protocol.

### 2.6 RNA extraction, RT‐PCR, and quantitative real‐time PCR

Total RNAs were extracted from cell lines and tissues using TRIzol reagent (Invitrogen, USA), and cDNAs were synthesized with Superscript II reverse transcriptase (Invitrogen) according to the manufacturer's protocol. Quantitative real‐time PCR (qPCR) was conducted to detect *P4HB*, *PHGDH*, *MAFbx*, *MURF1*, *Myod1* and *Bcl‐2* mRNA level using the SYBR Premix Ex TaqTM II (Tli RNase H Plus) kit (TaKaRa, Japan) and the Bio‐Rad (USA) machine. The small subunit ribosomal RNA (18sRNA) and glyceraldehyde 3‐phosphate dehydrogenase (GAPDH) were used as internal normalization references for mRNAs, respectively.

The following specific primers were used:

- hP4HB forward, 5′‐CACTGCAAACAGTTGGCTCC‐3′;
- hP4HB reverse, 5′‐CCGTTGTAATCAATGACCGT‐3′.
- hGAPDH forward, 5′‐TGTTGCCATCAATGACCCCTT‐3′;
- hGAPDH reverse, 5′‐CTCCACGACGTACTCAGCG‐3′.
- mP4HB forward, 5′‐GTCAACTGGCTGAAGAAACGC‐3′;
- mP4HB reverse, 5′‐CGCTTGAGTCCACCAAGGAC‐3′.
- mPHGDH forward, 5′‐TACCACAGGCTTGCTGAATGA‐3′;
- mPHGDH reverse, 5′‐GAGCACAGTTCACTACTCGCA‐3′.
- mMAFbx forward, 5′‐CAGCTTCGTGAGCGACCTC‐3′;
- mMAFbx reverse, 5′‐GGCAGTCGAGAAGTCCAGTC‐3′.
- mMURF1 forward, 5′‐GTGTGAGGTGCCTACTTGCTC‐3′;
- mMURF1 reverse, 5′‐GCTCAGTCTTCTGTCCTTGGA‐3′.
- mMyod1 forward, 5′‐GAGCAAAGTGAATGAGGCCTT‐3′;
- mMyod1 reverse, 5′‐CACTGTAGTAGGCGGTGTCGT‐3′.
- mBcl‐2 forward, 5′‐GTCGCTACCGTCGTGACTTC‐3′;
- mBcl‐2 reverse, 5′‐CAGACATGCACCTACCCAGC‐3′.
- mIL‐6 forward, 5′‐ TAGTCCTTCCTACCCCAATTTCC ‐3′;
- mIL‐6 reverse, 5′‐ TTGGTCCTTAGCCACTCCTTC‐3′.
- mCRP forward, 5′‐ TTCCCAAGGAGTCAGATACTTCC ‐3′;
- mCRP reverse, 5′‐ TCAGAGCAGTGTAGAAATGGAGA ‐3′.
- 18sRNA forward, 5′‐CAGCCACCCGAGATTGAGCA‐3′;
- 18sRNA reverse, 5′‐TAGTAGCGACGGGCGGTGT‐3′.
- mGAPDH forward, 5′‐CATGGCCTTCCGTGTTCCTA‐3′;
- mGAPDH reverse, 5′‐CCTGCTTCACCACCTTCTTG‐3′.

### 2.7 Western blot

The assays were performed as previously described^15^, using anti‐P4HB (Sigma, SAB1406213; Proteintech, 11245‐1‐AP), anti‐PHGDH (Proteintech, 14719‐1‐AP), anti‐MURF1 (Proteintech, 55456‐1‐AP), anti‐ALIX (Proteintech, 12422‐1‐AP), anti‐FLOT‐1 (Proteintech, 15571‐1‐AP), anti‐TSG101 (Proteintech, 14497‐1‐AP), anti‐LAMIN A/C (Proteintech, 10298‐1‐AP), anti‐albumin (Proteintech, 66051‐1‐Ig), anti‐PAX7 (Proteintech, 20570‐1‐AP), anti‐V5‐tag (Proteintech, 14440‐1‐AP), anti‐Bcl‐2 (Proteintech, 12789‐1‐AP, 60178‐1‐Ig; Cell Signalling Technology, #3498), anti‐HA‐tag (Proteintech, 66006‐1‐Ig; Cell Signalling Technology, #3724), anti‐cleaved PARP (Cell Signalling Technology, #9542), anti‐cleaved caspase‐3 (Cell Signalling Technology, #9664), anti‐cleaved caspase‐8 (Cell Signalling Technology, #8592; Sigma, HPA001302), anti‐Bax (Cell Signalling Technology, #2772), anti‐Cytochrome *c* (Cell Signalling Technology, #11940), anti‐pro caspase‐3 (Enzo Life Sciences, ADI‐AAP‐113‐F) anti‐β‐actin (Sigma, A5316), anti‐Flag‐tag (Sigma, F3165), anti‐LC3 (Sigma, L8918), anti‐MHC (Sigma, M4276); anti‐β‐tubulin (Sigma, T5201), anti‐GST (Santa Cruz Biotechnology, sc‐138), and anti‐GAPDH (Santa Cruz Biotechnology, sc‐25778) antibodies. For quantitative analysis, the membranes were scanned with the ImageQuant LAS 4000 (GE Healthcare Life Sciences) and the integrated density was measured using the software ImageJ (NIH).

### 2.8 Co‐immunoprecipitation

Cells were lysed with buffer (pH 7.6: 20 mM Tris/HCl, 150 mM NaCl, 20 mM KCl, 1.5 mM MgCl_2_, 0.8% NP‐40) added with Protease Inhibitor Cocktail (Roche). The extracts were incubated at 4°C overnight with Protein A/G Sepharose beads, which were preconditioned with antibodies or with anti‐FLAG M2 Magnetic Beads (Sigma, M8823). The beads were washed six times using ice‐cold lysis buffer. The immunoprecipitates were analyzed by immunoblotting.

For GST pull‐down assay, extracted Flag‐P4HB protein was incubated with GST or GST‐PHGDH fusion protein (Generay Biotech Co. Ltd., Shanghai, China) and glutathione Sepharose beads at 4°C overnight. Expression of GST fusion proteins was confirmed by SDS PAGE and Coomassie Blue staining.

For in vitro ubiquitination assay, indicated plasmids were transfected into C2C12 myoblasts for 36 h. The cells were incubated with 10 μM MG132 for 6 h and then harvested in lysis buffer. The supernatant was incubated with Protein A/G Sepharose beads at 4°C overnight. The immunoprecipitates were analyzed by immunoblotting.

### 2.9 Mass spectrometry

Proteins were extracted from GA muscle of YES2‐bearing mice. The extracts were incubated with Protein A/G Sepharose beads, which were preconditioned with anti‐P4HB (Proteintech, 11245‐1‐AP). The immunoprecipitates were subjected to SDS‐PAGE and visualized by silver staining. The differential band was digested for LC‐MS/MS analysis to identify proteins from the samples. The LC‐MS/MS analysis was carried out by CapitalBio Co. Ltd (Beijing, China).

### 2.10 Fluorescence microscopy study

Internalization of labelled EVs. EVs were labelled with PKH67 (Sigma) according to manufacturer's protocol. C2C12 myoblasts were seed in a 12‐well plate. After 12 h, cells were treated with 5 μg PKH67‐labelled YES2‐EVs for 4 h at 37°C. cells were then fixed with 4% paraformaldehyde (PFA) and incubated in 1% BSA/10% serum/0.1 M glycine in 0.1% PBS‐Tween for 1 h to block nonspecific protein‐protein interactions. Cells were incubated with anti‐GAPDH antibody (Proteintech, 10494‐1‐AP, 1:100). Nuclei were detected by DAPI. Images were collected using Laser‐scanning confocal microscope (Leica Microsystems Heidelberg GmbH, Am Friedensplatz 3, Germany).

To determine the diameter of myotubes in vitro, C2C12 myotubes were fixed by 4% PFA and incubated in 1% BSA/10% serum/0.1 M glycine in 0.1% PBS‐Tween for 1 h to block nonspecific protein‐protein interactions. Myotubes were incubated with anti‐MHC (Sigma, M4276, 1:100) diluted in 5% BSA overnight at 4°C. Images were collected using Laser‐scanning confocal microscope (Leica Microsystems Heidelberg GmbH, Am Friedensplatz 3, Germany). Images were captured by fluorescence microscope (Leica) and the diameter of myotubes was measured by Image J.

To examine the activation of satellite cells, satellite cells were incubated with EVs from KYSE150 cells with stable P4HB overexpression for 24 h and EdU reagent following the manufacturer's instructions. Satellite cells were fixed by 4% PFA and incubated in 1% BSA/10% serum/0.1 M glycine in 0.1% PBS‐Tween for 1 h to block nonspecific protein‐protein interactions and incubated with self‐renewing factor anti‐PAX7 (Proteintech, 20570‐1‐AP, 1:100) diluted in 5% BSA overnight at 4°C. Nuclei were detected by DAPI. Images were collected using Laser‐scanning confocal microscope (Leica Microsystems Heidelberg GmbH, Am Friedensplatz 3, Germany).

Colocalization assay. C2C12 myoblasts were seeded in a 12‐well plate. After 12 h, cells were then fixed with 4% PFA and incubated in 1% BSA/10% serum/0.1 M glycine in 0.1% PBS‐Tween for 1 h to block nonspecific protein‐protein interactions. Cells were incubated with corresponding antibodies followed by Alexa Fluor 488 and Alexa Fluor 594‐secondary antibody (ZSGB‐BIO). Nuclei were detected by DAPI. Images were collected using Laser‐scanning confocal microscope (Leica Microsystems Heidelberg GmbH, Am Friedensplatz 3, Germany).

### 2.11 Proliferation assay

Cell proliferation assay was measured via the xCELLigence Real‐Time Cell Analyzer (RTCA)‐MP system (Acea Biosciences/Roche). 100 μl of culture medium was added in each well of E‐Plate 96 (Roche Applied Science) to obtain baseline. 800 cells in 100 μl of culture medium with indicated EVs were seeded in E‐Plate 96, which was put in RTCA‐MP device at 37°C with 5% CO_2_. The Cell Index was automatically recorded every 15 min and the data were shown as cell growth curve.

### 2.12 Triglyceride (TG) measurement

Epididymal white adipose tissues (eWAT) were taken from mice immediately after euthanasia. Triglyceride measurement was conducted using commercial TG assay kits (Nanjing Jiancheng Biological Product Co. Ltd, China; A110‐1‐1) according to the manufacturer's instructions and at last the absorbance OD (510 nm) was detected on a BIOTEK microplate reader.

### 2.13 ELISA assay

Serum levels of Interleukin‐6 (IL‐6) and C‐reaction protein (CRP) were measured on serum samples from mice with and without CCF642 inhibitor treatment using mouse IL‐6 and CRP ELISA kit (Neobioscience, China) according to the manufacturer's instructions.

### 2.14 TUNEL staining

TUNEL staining was conducted using the in situ colorimetric TUNEL apoptosis assay kit (Beyotime, China), according to the manufacturer's protocol. Briefly, the paraffin sections were deparaffinized and washed. Proteinase K without DNase was added and calibrated at 37°C for 15–30 min. After washing with PBS, we used 3% H_2_O_2_ (configured with PBS) for incubating the sections at room temperature for 20 min. After inactivated the endogenous peroxidase of the slices, the sections were washed with PBS. Then, the samples were labelled with biotin and stained according to the instructions.

### 2.15 Flow cytometry

C2C12 myoblasts (2×10^5^ cells per well) were seeded in 6‐well plates and allowed to reach 60% confluence. The culture medium was removed and replaced with fresh culture medium containing EVs (10 μg) for 24 h. Cells were collected and washed with cold 1 × PBS. For analysis of apoptosis, cells were incubated with Annexin V and propidium iodide (PI) using an apoptosis analysis kit (Neobioscience, China).

### 2.16 Inhibitor of P4HB treatment assay in vitro and in vivo

For apoptosis assay, 2 × 10^5^ C2C12 myoblasts were seeded into 6‐well plates and allowed to reach 60% confluence, then replaced with fresh medium addition with concentrations of 0, 0.1 μM, 1 μM, 3 μM, 5 μM CCF642 concomitant with 35 μM cisplatin or 0.1% DMSO vehicle alone. After 24 h of incubation at 37°C, cells were collected, washed with 1×PBS and incubated with Annexin V and propidium iodide (PI) using an apoptosis analysis kit (Neobioscience, China).

For in vivo study, 3×10^6^ YES2 cells (100 μl) were injected subcutaneously into the right flanks of BALB/c nude mice. Body weight and tumour size were measured twice a week. When the average tumour volume reached 50 mm^3^, tumour‐bearing mice were randomly divided into two groups (*n* = 10 per group). Mice were intraperitoneally (i.p.) administrated CCF642(10 mg/kg) concomitant with cisplatin (5 mg/kg) three times a week for 4 weeks. Control mice were given albumin with cisplatin. Mice were euthanized to measure the body weight, GA muscle weight and tumour weight. Tissues were harvested and embedded in paraffin for H&E analysis.

### 2.17 Statistical analysis

The data were presented as means ± s.e.m for at least three biological replicates. The differences between experimental groups were assessed using two‐tailed, unpaired Student's *t*‐test for group comparisons, the two‐tailed Pearson *χ* ^2^ test was used to analyze the association of P4HB expression and clinicopathologic parameters, and Kruskal‐Wallis test was used to analyze the inflammation events using the GraphPad Prism 5 and IBM SPSS Statistics 19 software. ns, not significant; *, *P* < 0.05; **, *P* < 0.01; ***, *P* < 0.001.

## 3 RESULTS

### 3.1 Development of an ESCC‐induced cachexia mouse model

**FIGURE 1:**
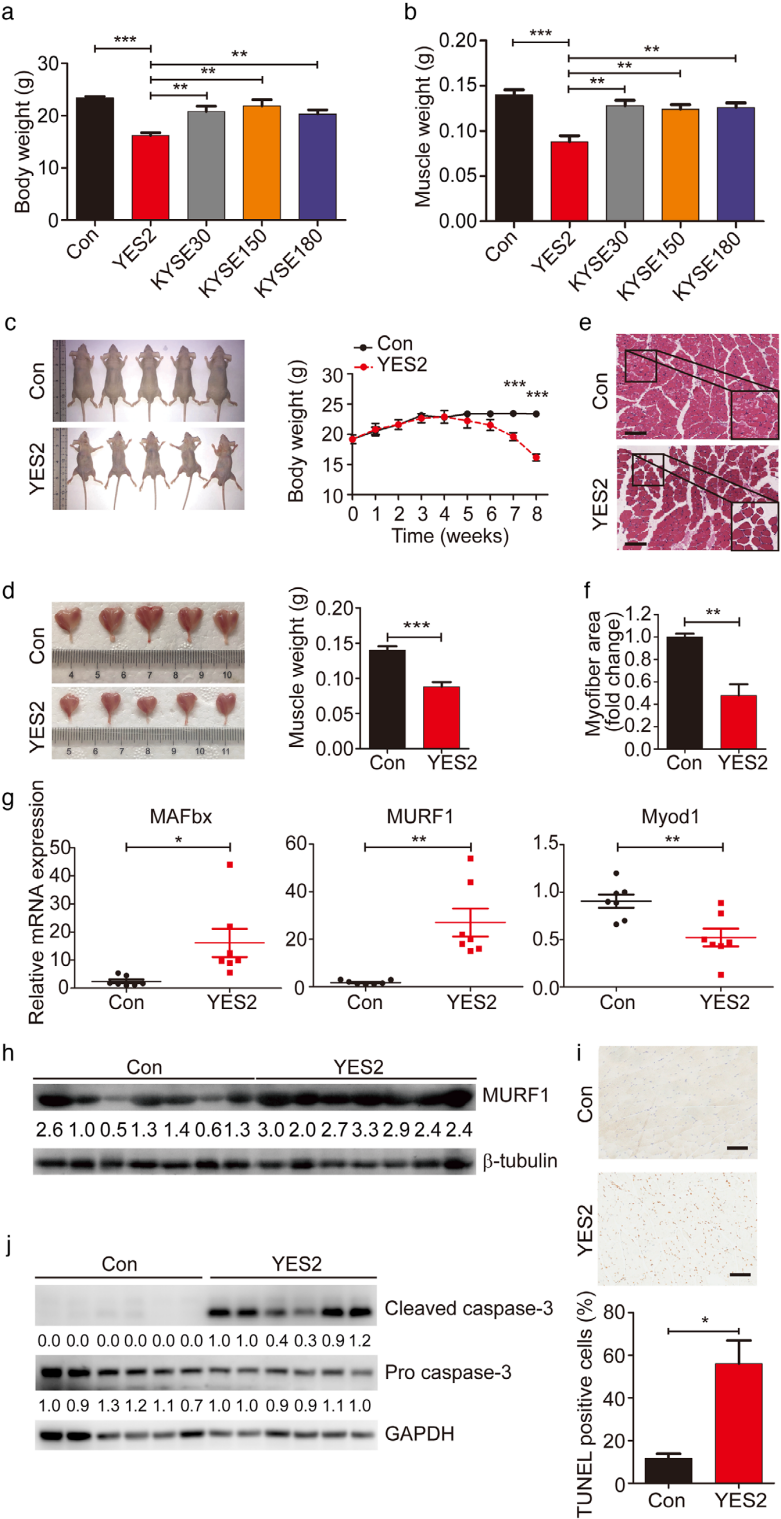
Development of an ESCC‐induced cachexia mouse model. (a) Body weight analysis of xenograft‐bearing Balb/c nude mice subcutaneously implanted with YES2 (n = 5), KYSE30 (n = 5), KYSE150 (n = 5) and KYSE180 (n = 5) and of respective non‐tumour‐bearing control (Con) mice. (b) GA muscle weight analysis of xenograft‐bearing Balb/c nude mice. (c) Mouse images (left) and body weight changes (right) of xenograft‐bearing Balb/c nude mice subcutaneously implanted with YES2 cells (n = 5) and of respective non‐tumour‐bearing control (Con; n = 5) mice. Mouse images were taken at 8 weeks after YES2 injection. (d) GA muscle images (left) and muscle weight analysis in xenograft‐bearing Balb/c nude mice (n = 5) and non‐ tumour ‐bearing control (Con; n = 5) mice. (e) Representative micrographs of H&E histology of GA muscle in YES2‐bearing mice, relative to non‐tumour‐bearing control mice (n = 5). Scale bars, 150 μm. (f) Quantification of the myofiber cross‐sectional areas in YES2‐bearing mice versus non‐bearing mice. (g) mRNA expression of muscle atrophy markers, MAFbx, MURF1 and Myod1, in GA muscle. n = 7 for Con mice and n = 7 mice for YES2‐bearing mice. (h) Western blot of MURF1 protein in GA muscle from non‐tumour‐bearing control mice (n = 7) and YES2‐bearing mice (n = 7). (i) The apoptosis of GA muscle in YES2‐bearing mice (n = 5), relative to non‐tumour‐bearing control mice (n = 5), was measured by terminal TUNEL assay and quantification of percentage of TUNEL positive cells. Scale bars, 100 μm. (j) Western blot analysis of cleaved caspase‐3 protein and pro caspase‐3 protein in GA muscle from non‐ tumour ‐bearing control mice (n = 6) and YES2‐bearing mice (n = 6)

To investigate the mechanisms underlying muscle wasting driven by ESCC in vivo, we first developed an ESCC model of cachexia through subcutaneous implantation of four human ESCC cell lines (YES2, KYSE30, KYSE150 and KYSE180) into nude mice. In comparison with the body weight of non‐tumour‐bearing mice (control group), mice implanted with YES2 cells exhibited decreased body weight (Figure 1, a and c, and Fig. S1A, *P* < 0.0001), while xenograft tumour had no effects on growth and food intake of YES2‐bearing mice (Fig. S1B and C).

Furthermore, wasting of gastrocnemius (GA) muscle and reduction of muscle weight were observed in YES2‐bearing mice (Figure 1, b and d, *P *= 0.0003 in Figure 1d). Histopathological analysis of GA muscle sections revealed that YES2‐bearing mice maintained marked muscle wasting compared to non‐tumour‐bearing mice (Figure 1e) and myofiber cross‐sectional areas in GA muscle were much smaller in YES2‐bearing mice than that in non‐tumour‐bearing mice (Figure 1f, *P *= 0.0011). Moreover, the mRNA levels of muscle wasting marker *MAFbx* (*FBXO32*, *Atrogin1*) and ubiquitin E3 ligases *MURF1* were significantly upregulated in GA muscle of YES2‐bearing mice. In contrast, the transcription of myogenesis‐related gene *Myod1* was lower in GA muscle of YES2‐bearing mice than that in non‐tumour‐bearing mice (Figure 1g, *P *= 0.02, *P *= 0.001, *P *= 0.0067, respectively). Consistently, the protein levels of MURF1 were elevated in GA muscle of YES2‐bearing mice, relative to non‐tumour‐bearing mice (Figure 1h). Besides that, to explore whether muscle wasting was specific in GA muscles or it was a more generalized process, we examined the mRNA levels of *MAFbx* and *MURF1* in other muscle types including tibialis anterior (TA), quadriceps, soleus and cardiac muscles, and found that *MAFbx* and *MURF1* expression was consistently elevated in all of these muscles when compared YES2‐bearing mice with non‐tumour‐bearing mice (Fig. S1E). Histopathological analysis revealed that myofiber cross‐sectional areas in all of these muscle sections were much smaller in YES2‐bearing mice than that in non‐tumour‐bearing mice (Fig. S1F). Taken together, these data demonstrated that YES2 cells systemically induced cachexia in muscle tissue groups including multiple fibre types.

Considering cachexia can induce apoptosis leading to muscle wasting (Bowen et al., 2017), we thus performed TUNEL staining assay and observed increased apoptotic activity in muscle sections of YES2‐bearing mice (Figure 1i). In consistent with this observation, cleaved caspase‐3, an apoptosis‐related marker, was upregulated in the cachectic GA muscle from YES2‐bearing mice (Figure 1j), suggesting that activation of apoptotic pathway contributed to muscle wasting in this mouse model. These observations were also recurrent in a mouse cachexia model of Lewis lung carcinoma (LLC) (Fig. S2), a well‐recognized cachexia‐inducible mouse model (Acharyya et al., 2005). Together, these results indicated that, we have successfully generated an ESCC xenograft mouse model of cachexia using YES2 cell line, which could be beneficial for the following cachexia research in ESCC.

### 3.2 Identification and characterization of EVs derived from ESCC

**FIGURE 2:**
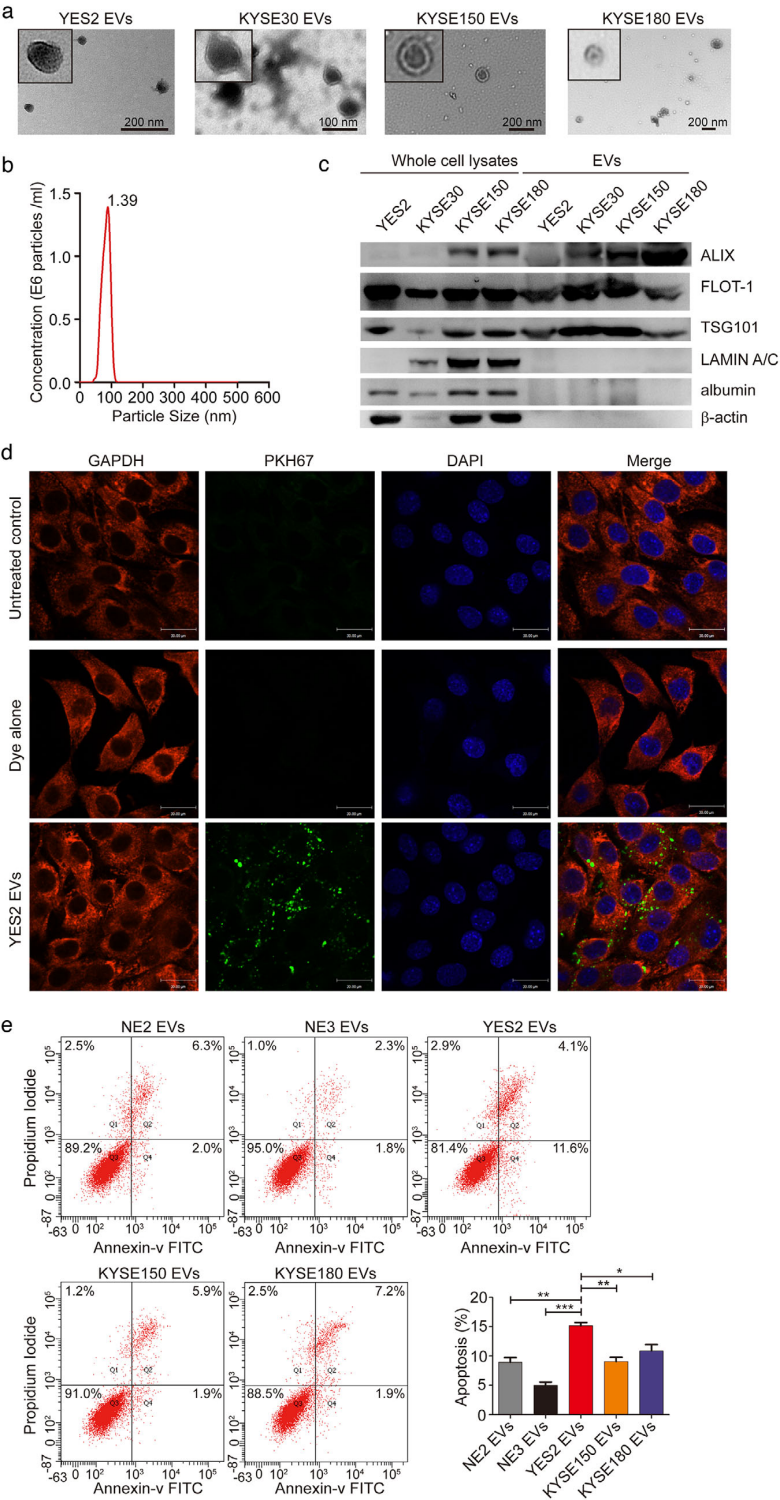
Identification and characterization of EVs derived from ESCC. (a) Representative transmission electron microscopy image of ESCC cell line‐derived EVs (YES2, KYSE30, KYSE150 and KYSE180). (b) Nanoparticle tracking analysis (NTA) indicated the size distribution and concentration of EVs secreted by YES2 cells. (c) Western blot showed expression of EV markers (ALIX, FLOT‐1, and TSG101) in the whole cell lysates and EVs derived from YES2, KYSE30, KYSE150 and KYSE180 cells. LAMIN A/C, albumin and β‐actin were used as non‐exosomal markers. The amount of EVs and cells loaded was normalized to the amount of total proteins. (d) Confocal microscopy images of the internalization of YES2 EVs labelled with 2 nM PKH67 dye in C2C12 myoblasts. Dye alone group was used as a positive control. Untreated control group was used as a negative control. Scale bars, 30 μm. (e) For apoptosis analysis, C2C12 myoblasts were treated with NE2, NE3, YES2, KYSE150 and KYSE180 EVs (10 μg) for 24 h. The cells were collected and stained with annexin V and PI

EVs from cancer cells encapsulating a variety of functional proteins can communicate with target cells by internalization and then regulate their physiological functions (Costa‐Silva et al., 2015; Sagar et al., 2016). This inspired us to examine whether ESCC‐derived EVs containing some cargos play the roles in muscle wasting in the cachexia model. We first isolated EVs from the conditioned medium of ESCC cell lines. Electron microscopy and NanoSight analysis showed that vesicles isolated were EVs with a diameter of 30–150 nm (Figure 2, a and b), which was confirmed by the detection of EV‐specific protein markers, ALIX, FLOT‐1, and TSG101. The purity of the isolated EVs was confirmed by Lamin A/C, albumin and β‐actin, which are absent in EVs (Figure 2c). Next, we tested whether these ESCC‐derived EVs could be internalized by muscle cells in vitro using C2C12 murine myoblasts. Isolated EVs were labelled with the fluorescent dye PKH67 and added into the culture medium of C2C12 myoblasts. After 4 h incubation, spots with green fluorescence presented in the cytoplasm of C2C12 myoblasts (Figure 2d). This result suggested that C2C12 myoblasts have efficiently uptaken ESCC‐EVs. Then we assessed the effects of ESCC cell lines‐derived EVs on C2C12 myoblasts in vitro using flow cytometry based apoptotic assay. Compared with other cell lines, YES2‐EVs induced apoptosis in C2C12 myoblasts (Figure 2e), consistent with the specific observation of cachexia in the YES2‐bearing mice. Together, these data highlighted the importance of considering the transmitting role of EVs during the development of ESCC‐induced cachexia.

### 3.3 P4HB is identified as a crucial mediator of muscle wasting in vitro and in vivo

**FIGURE 3:**
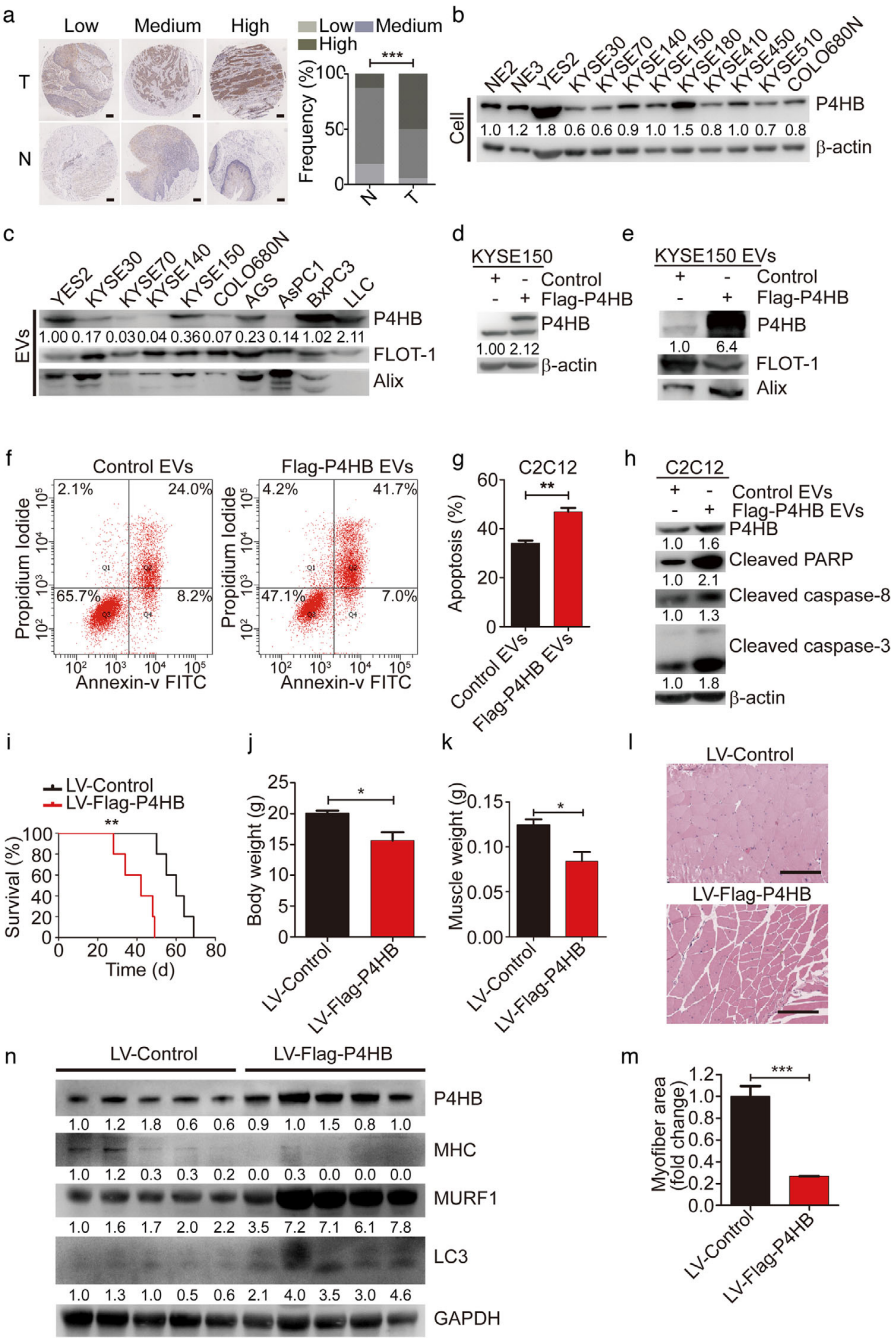
P4HB is identified as a crucial mediator of muscle wasting in vitro and in vivo. (a) Micrographs after immunohistochemistry against P4HB in ESCC tissues (T) and matched adjacent normal tissues (N). Scale bars, 200 μm. The frequency of P4HB expression levels in 77 pairs of human ESCC tissues and matched adjacent normal tissues. (b) The P4HB protein levels in two immortalized esophageal epithelial cell lines (NE2 and NE3) and 10 ESCC cell lines were analyzed by immunoblotting. (c) The P4HB levels in EVs released from ESCC cell lines and other types of cancer cells known as cachexia‐induced cancer cells (AGS, AsPC1, BxPC3, LLC). (d) Overexpressed P4HB protein levels after transfecting the Flag‐P4HB plasmid in KYSE150 cell lines. (e) The P4HB levels in EVs secreted by KYSE150 transfected with Flag‐P4HB plasmid. (f and g) For apoptosis analysis, C2C12 myoblasts were treated with EVs (10 μg) for 24 h derived from KYSE150 cells transfected with Flag‐P4HB plasmid induced by 35 μM cisplatin. (h) Western blot analysis of apoptotic markers in C2C12 myoblasts treated with EVs (10 μg) for 24 h derived from KYSE150 cells transfected with Flag‐P4HB plasmid in combination with 35 μM cisplatin. (i) Kaplan–Meier survival curves of nude mice subcutaneously implanted with KYSE150 cells with stable P4HB overexpression (n = 5), relative to P4HB negative control mice (n = 5). (j and k) Body weight and GA muscle weight changes in KYSE150 implanted mice (n = 5 for each group). (l) Representative micrographs of H&E histology of GA muscle in KYSE150 implanted mice (n = 5 for each group). Scale bars, 150 μm. (m) Quantification of the average myofiber cross‐sectional areas of KYSE150 implanted mice (n = 5 for each group). (n) Western blotting in vivo of MHC, MURF1 and LC3 protein in GA muscle from mice implanted with KYSE150 cells with stable P4HB overexpression (n = 5), relative to P4HB negative control mice (n = 5)

We then wanted to determine which cargo contained in EVs contributed to the ESCC‐associated cachexia. P4HB was reported to be amplified with a high frequency (9.09%) in our ESCC cases (Cheng et al., 2016; Song et al., 2014) and multiple types of cancer (Fig. S3A), and its overexpression could predict poor survival of patients with ESCC (Fig. S3B and C). Previous epigenome‐wide association and functional studies unveiled the strong association of P4HB with body mass index and proliferation/apoptosis of smooth muscle cells (Ping et al., 2017; Wahl et al., 2016). We thus selected P4HB for further ESCC‐associated cachexia analysis, and examined protein expression of P4HB in 77 pairs of human ESCC tissues and matched adjacent normal tissues utilizing immunohistochemical assay. Consistently, P4HB was expressed at higher levels in ESCC tissues than in matched adjacent normal tissues (Figure 3a, *P* < 0.0001). We further investigated the association of P4HB expression with clinicopathological features of patients with ESCC. Statistical analyses showed that P4HB expression level had significant correlation with tumour differentiation of ESCC (Table S1, *P *= 0.016).

Next, we carried out western blotting in ESCC cell lines and normal oesophageal cell lines NE2 and NE3. As showed in Figure 3b, the protein levels of P4HB were significantly higher in YES2 than in normal oesophageal cell lines and other ESCC cell lines. In addition, we observed higher P4HB levels in EVs released from YES2 cells and other well‐known cachexia‐inducible cancer cells, including AGS (human gastric adenocarcinoma), BxPC3 (human pancreatic adenocarcinoma), and LLC (mouse Lewis lung carcinoma) (Fukawa et al., 2016; He et al., 2014; Yang et al., 2019; Zhang et al., 2017) compared to other ESCC cell lines (Figure 3c). We also observed elevated P4HB protein level in GA muscle of YES2‐bearing mice (Fig. S1D). These results suggested that dysregulation of P4HB was involved in the pathology of cachexia.

To confirm this hypothesis, we overexpressed Flag‐P4HB in KYSE150 or knocked down P4HB transcription in YES2 (Figure 3d and Fig. S4A). Overexpression of Flag‐P4HB significantly increased P4HB levels in KYSE150‐EVs (Figure 3e).

Cisplatin is one of the commonly used first‐line chemotherapeutic agents for ESCC treatment (Vermorken et al., 2007). However, cancer treatment, mainly chemotherapy can lead to cancer cachexia and muscle wasting (Bricon et al., 1995), which further reduced treatment tolerance and survival of patients (Johns et al., 2013). P4HB‐overexpressed KYSE150‐EVs strikingly increased the apoptosis rates of C2C12 myoblasts induced by cisplatin (Figure 3, f and g, *P *= 0.0024). Conversely, treatment with P4HB‐knockdown YES2‐EVs (Fig. S4B) significantly inhibited the rates of apoptosis in C2C12 myoblasts induced by cisplatin (Fig. S4C and D, *P *= 0.0002, *P *= 0.0004). We further transfected C2C12 myoblasts with P4HB plasmid to mimic the delivery through artificial EVs packaging specific P4HB protein. Expectedly, treatment with P4HB plasmid resulted in enhanced rates of cisplatin‐induced apoptosis (Fig. S5A, *P *= 0.016).

We also stably overexpressed P4HB in KYSE150 cells using lentiviral vector and knocked down P4HB in YES2 cells with lentiviral shRNA. There was the same tendency of apoptosis in C2C12 myoblasts as treatment with transient transfection (Fig. S5B and C, *P *= 0.0357, *P *= 0.006, respectively). To explore whether EVs played a critical role in this effect, EVs production was reduced through the pharmacological inhibition of neutral sphingomyelinase‐2 (nSMase) with GW4869. As shown in Fig. S5D, EVs from KYSE150 cells with stable P4HB overexpression treated with GW4869 failed to increase the apoptosis rates of C2C12 myoblasts induced by cisplatin. Additionally, GW4869 treatment inhibited the release of EVs, which packaged P4HB protein, resulting in the decreased apoptotic rates of C2C12 myoblasts (Fig. S5E). These data confirmed that EVs containing P4HB derived from tumour played a crucial role in regulating muscle wasting.

Apart from above, we found that treatment with KYSE150‐EVs with stable P4HB overexpression suppressed cell proliferation. In contrast, treatment with YES2‐EVs with stable P4HB depletion restored cell growth of C2C12 myoblasts (Fig. S5F and G).

The protein levels of apoptosis‐related markers were analyzed by immunoblotting. We observed that KYSE150‐EVs with P4HB overexpression resulted in the upregulation of cleaved PARP, caspase‐3 and caspase‐8 in C2C12 myoblasts (Figure 3h). In contrast, the protein levels of cleaved PARP, caspase‐3 and caspase‐8 were reduced by treatment with YES2‐EVs with P4HB depletion (Fig. S4E). These findings suggested that elevated P4HB levels in EVs induced apoptosis in C2C12 myoblasts through activating extrinsic apoptosis pathway by upregulating cleaved caspase‐8 level.

Furthermore, the role of P4HB in myoblast apoptosis was also investigated in another cachexia‐inducible cancer cell line LLC. Higher P4HB levels were also observed in cachexia‐inducible cells, including AGS, BxPC3 and LLC (Fig. S6A). Knockdown of P4HB in LLC cells (Fig. S6B) decreased P4HB levels in LLC‐EVs (Fig. S6C), which strikingly inhibited the apoptosis rates of C2C12 myoblasts induced by cisplatin combined with LLC‐EVs (Fig. S6D and E, *P =* 0.0056, *P =* 0.0025, respectively). In consistence with this observation, the protein levels of cleaved PARP, caspase‐3 and caspase‐8 were reduced by treatment with P4HB‐depleted LLC‐EVs (Fig. S6F). These findings indicated that P4HB might be a critical factor of inducing cachexia of pan‐cancer types.

Moreover, P4HB‐overexpressed EVs treatment resulted in activation of satellite cells, which was associated with cancer cachexia (Fig.S7A). In differentiated C2C12 myotubes induced by cisplatin, treatment using KYSE150‐EVs with stable P4HB overexpression reduced myotube diameter, decreased expression level of myosin heavy chain (MHC), elevated levels of MURF1 and an autophagy marker, LC3 (Fig. S7B‐D). The proportion of TUNEL‐positive cells was significantly increased in C2C12 myotubes treated with LV‐Flag‐P4HB EVs (Fig. S7E). Taken together, P4HB could promote muscle atrophy in vitro.

To evaluate the role of elevated P4HB expression in ESCC‐induced muscle wasting *in vivo*, nude mice were subcutaneously implanted with KYSE150 cells stably overexpressing P4HB via lentiviral vectors (KYSE150 LV‐Flag‐P4HB). Kaplan–Meier survival curves showed that KYSE150 LV‐Flag‐P4HB cells accelerated the mortality of mice compared to mice injected with KYSE150 LV‐Control cells (Figure 3i *P *= 0.0018). Overexpressed P4HB expedited the development of muscle wasting as measured by body weight (Figure 3j, *P *= 0.024) and GA muscle weight (Figure 3k, *P *= 0.012). Histopathological analysis on GA muscle cross‐sectional areas showed a significant shrinkage of myofibers in the KYSE150 LV‐Flag‐P4HB group (Figure 3l). Most myofibers in the KYSE150 LV‐Flag‐P4HB group had much smaller cross‐sectional areas than those in the KYSE150 LV‐Control group (Figure 3m, *P* < 0.0001). Additionally, KYSE150 LV‐Flag‐P4HB cells triggered ESCC‐induced cachectic response measured by decreased level of MHC, elevated levels of MURF1 and LC3 protein (Figure 3n). Collectively, these results suggested that P4HB played a crucial role in inducing muscle wasting, which might be the potential cellular mechanism of regulating cachexia in vivo.

### 3.4 P4HB interacts with PHGDH and destabilizes PHGDH by increasing its ubiquitination level

**FIGURE 4:**
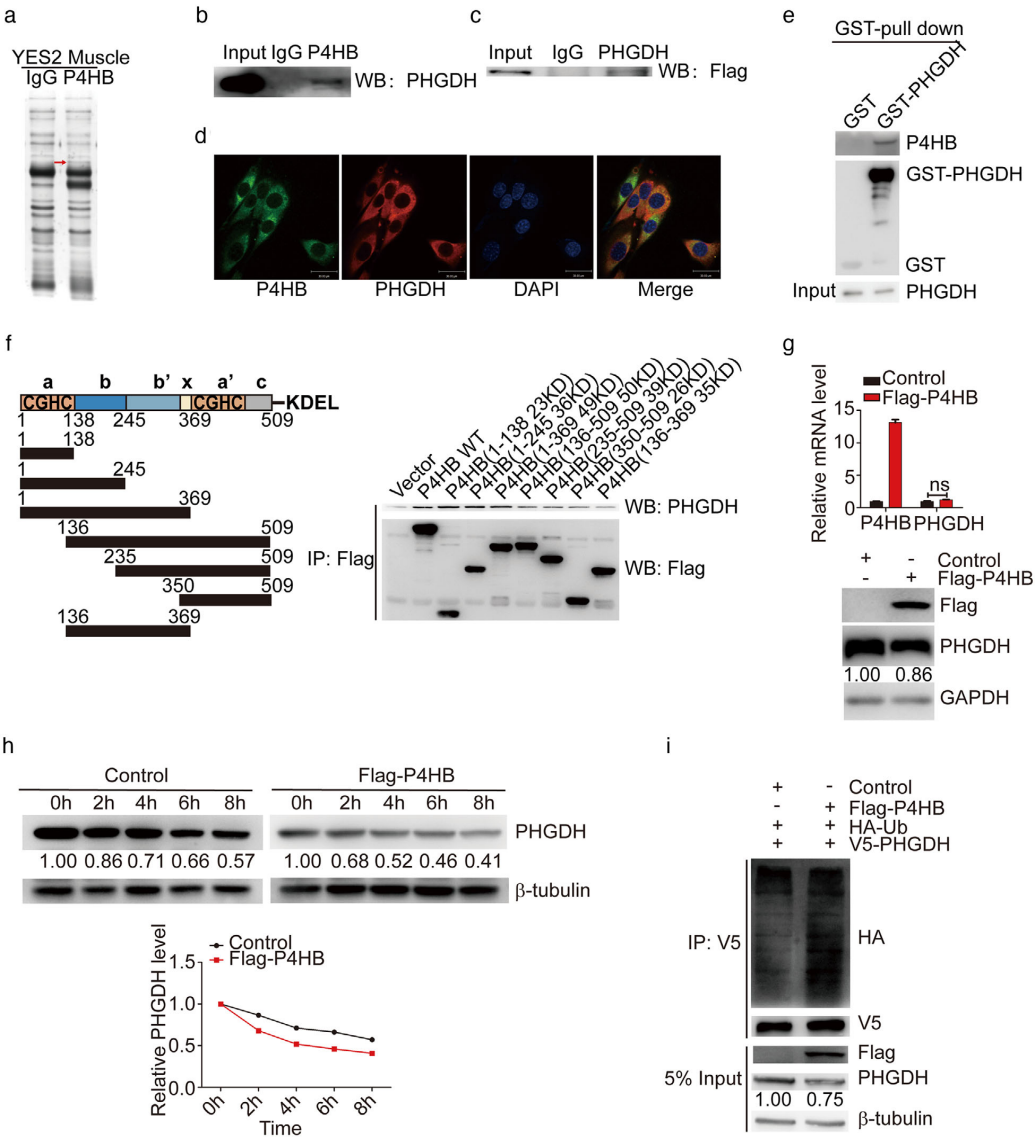
P4HB interacts with PHGDH and destabilizes PHGDH by increasing its ubiquitination level. (a) Identification of P4HB‐interacting proteins. Proteins extracted from GA muscle of YES2‐bearing mice were incubated with Protein A/G Sepharose beads conjugated with anti‐P4HB antibody, subjected to SDS‐PAGE and visualized by silver staining. The band indicated by the red arrow was subjected to mass spectrometry. (b) Endogenous PHGDH detected by the co‐immunoprecipitation assay with anti‐P4HB antibody in C2C12 myoblasts. The immunoglobulin G (IgG) was used as the control group. (c) Flag‐P4HB plasmid was transfected into C2C12 myoblast, and exogenous Flag‐tag was detected by the co‐immunoprecipitation assay with anti‐PHGDH antibody. (d) Immunofluorescence showed that endogenous PHGDH and P4HB colocalized in C2C12 myoblasts. Scale bars, 30 μm. (e) In vitro GST pull‐down assay to verify the interaction between P4HB and PHGDH. C2C12 myoblasts were transfected with GST alone or GST‐PHGDH. Retrieved proteins were evaluated with immunoblotting. GST‐immunocomplexes were boiled and subjected to immunoblotting analysis using P4HB antibody. (f) Left panel: A schematic diagram shows the structural domains of P4HB. Right panel: The control vector, Flag‐tagged wild type or truncated mutants of P4HB were transfected into C2C12 myoblasts. The cell lysates were subjected to the co‐immunoprecipitation assay by anti‐FLAG M2 Magnetic Beads and PHGDH expression was analyzed by immunoblotting. (g) P4HB plasmid was transfected into C2C12 myoblasts for 48 h and mRNA levels of P4HB and PHGDH were analyzed by RT‐qPCR assay (top panel). The protein levels of P4HB and PHGDH were analyzed by immunoblotting (bottom panel). (h) C2C12 myoblasts transfected with P4HB plasmid were treated with 100 μg/ml CHX and harvested at the indicated time point. The cell lysates were subjected to immunoblotting and PHGDH expression was quantified by ImageJ software. (i) HA‐Ub and V5‐PHGDH plasmid were co‐transfected with Flag‐P4HB plasmid in C2C12 myoblasts. After 36 h, cells were treated with 10 μM MG132 for 6 h. Anti‐V5 antibody was used to immunoprecipitate with exdogenous P4HB. Its ubiquitination level was assessed by anti‐HA antibody. 5% input of cell lysates was used to analyze the expression of P4HB and PHGDH

To uncover the mechanism underlying P4HB‐associated apoptosis, YES2 cells were subcutaneously implanted into nude mice. Skeletal muscle of these implanted mice was then used for immunoprecipitation coupled with mass spectrometry to identify P4HB‐interacting proteins (Figure 4a; Fig. S8A). Here, phosphoglycerate dehydrogenase (PHGDH) was selected for further analysis because it was a key mediator involved in the suppression of apoptotic response in melanoma cells (Ou et al., 2015). The interaction between P4HB and PHGDH was further confirmed by co‐immunoprecipitation assay in C2C12 myoblasts (Figure 4b and c). We examined the basal protein level of P4HB in C2C12 myoblasts and other tumour cells, indicating that P4HB was expressed in normal C2C12 myoblasts, in which the level of P4HB was lower than that in YES2 cells (Fig. S8B). Additionally, we also examined their subcellular localizations in C2C12 myoblasts by staining with P4HB and PHGDH antibodies, respectively. Confocal microscopy assay exhibited a colocalization of P4HB (bright green) and PHGDH (bright red) (Figure 4d). Moreover, we performed a glutathione S‐transferase (GST) affinity isolation assay to determine whether P4HB directly interacted with PHGDH. Obviously, P4HB was co‐purified with GST‐PHGDH (Figure 4e). These data indicate that P4HB directly interacts with PHGDH. To identify the potential region of P4HB responsible for its interaction with PHGDH, we generated seven P4HB truncated mutants (Gruber et al., 2006), and observed that wild‐type P4HB and the first domain a (aa 1–138) maintained their interaction with PHGDH (Figure 4f). Given that redox‐active domain a (aa 1–138) provides the ability to catalyze redox reactions (Alanen et al., 2003), it might be essential for their interaction and regulation.

To elucidate the function of P4HB on PHGDH regulation, we examined the expression levels of PHGDH in Flag‐P4HB‐overexpressed C2C12 myoblasts. The protein levels of PHGDH were decreased, however, *PHGDH* mRNA levels did not show any significant alterations (Figure 4g). Consistently, the levels of PHGDH protein, but not its mRNA level were dramatically increased by P4HB siRNA (Fig. S8C), indicating that P4HB regulated PHGDH expression at the post‐translational level.

We further examined whether P4HB regulated the stability of PHGDH protein using the cycloheximide (CHX) pulse‐chase assay. As shown in Figure 4h, P4HB overexpression strikingly accelerated PHGDH degradation. Conversely, knockdown of P4HB extended the half‐life period of PHGDH (Fig. S8D). Western blotting analysis revealed that PHGDH was mediated through ubiquitin‐dependent proteolytic pathway (Fig. S8E).

In addition, an in vitro ubiquitination assay revealed that Flag‐P4HB overexpression dramatically increased the poly‐ubiquitination level of PHGDH (Figure 4i); in contrast, the ubiquitination level of PHGDH was significantly reduced when P4HB was knocked down in C2C12 myoblasts (Fig. S8F). In conclusion, these findings indicated that a direct interaction between P4HB and PHGDH was mediated through redox‐active domain of P4HB, and overexpression of P4HB downregulated PHGDH by increasing ubiquitination level of PHGDH to accelerate its degradation.

### 3.5 Depletion of PHGDH induces apoptosis through reducing Bcl‐2 stability

**FIGURE 5:**
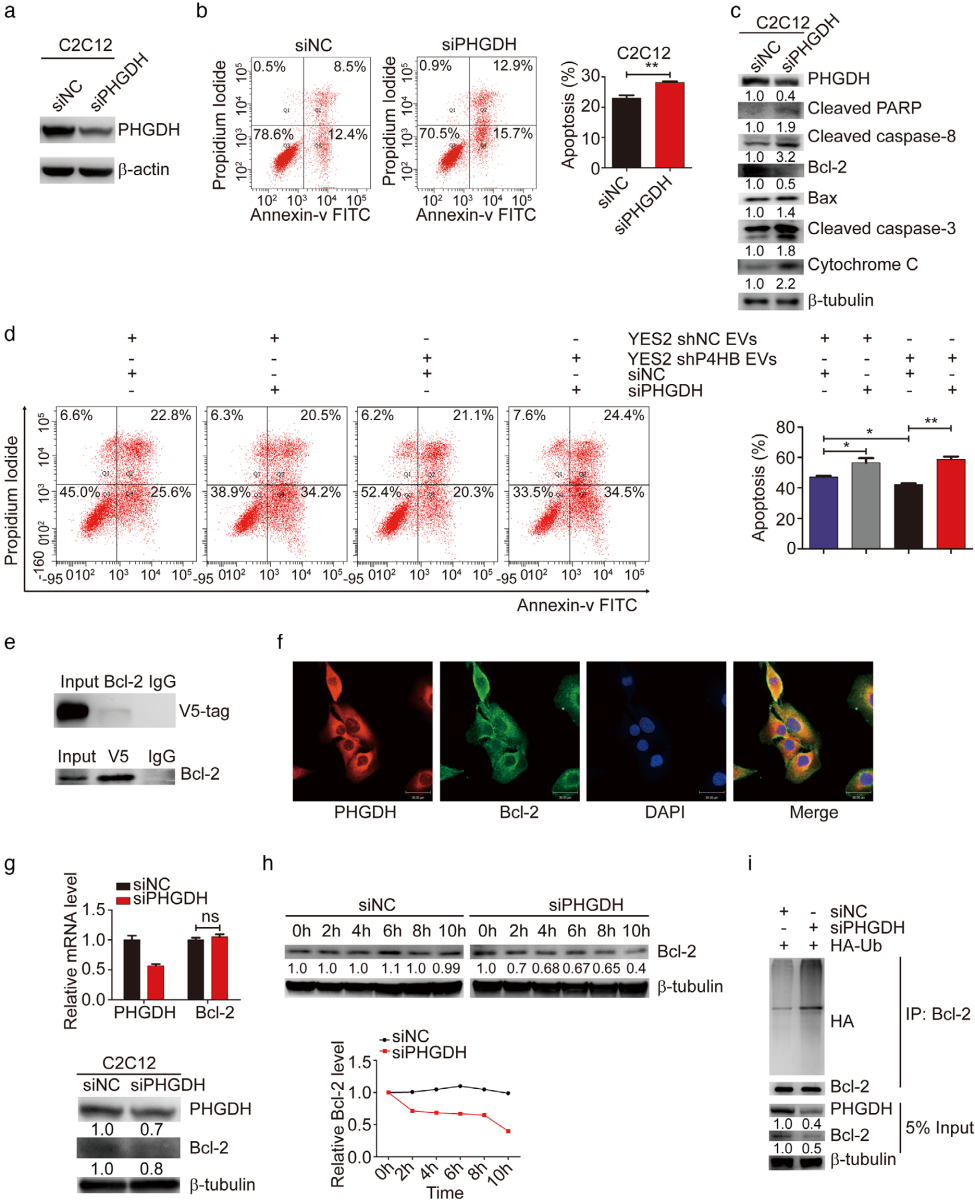
Depletion of PHGDH induces apoptosis through reducing Bcl‐2 stability. (a) PHGDH siRNA was transfected into C2C12 myoblasts for 48 h and protein levels of PHGDH were analyzed by immunoblotting. (b) The apoptosis assay in C2C12 myoblasts transfected with PHGDH siRNA induced by 35 μM cisplatin for 24 h. (c) Western blot analysis of apoptotic markers in C2C12 myoblasts transfected with PHGDH siRNA induced by 35 μM cisplatin for 24 h. (d) The assay of apoptosis in C2C12 myoblasts transfected with PHGDH siRNA in combination with stable P4HB‐depleted YES2‐EVs induced by 35 μM cisplatin for 24 h. Representative images (left panel) and quantitative analysis (right panel) of apoptotic rates. (e) V5‐PHGDH plasmid was transfected into 293T cells and V5‐tag was detected by the co‐immunoprecipitation assay with anti‐Bcl‐2 antibody. The immunoglobulin G (IgG) was used as the control group (top panel). V5‐PHGDH plasmid was transfected into 293T cells and endogenous Bcl‐2 was detected by the co‐immunoprecipitation assay with anti‐V5 antibody (bottom panel). (f) Immunofluorescence showed that endogenous PHGDH and Bcl‐2 colocalized in C2C12 myoblasts. Scale bars, 30 μm. (g) The mRNA and protein levels of PHGDH and Bcl‐2 in C2C12 myoblasts with PHGDH siRNA were respectively analyzed by RT‐qPCR assay (top panel) and immunoblotting assay (bottom panel). (h) 293T cells transfected with PHGDH siRNA were treated with 100 μg/ml CHX and harvested at the indicated time point. The cell lysates were subjected to immunoblotting and PHGDH expression was quantified by ImageJ software. (i) 293T cells were co‐transfected with HA‐Ub and PHGDH siRNA for 36 h and then cells were incubated with 10 μM MG132 for 6 h. Bcl‐2 ubiquitination level was assessed by immunoprecipitation

To examine whether PHGDH was involved in P4HB‐mediated apoptosis in C2C12 myoblasts, the protein level of PHGDH was knocked down using siRNA in C2C12 myoblasts (Figure 5a). Transfection of PHGDH siRNA in C2C12 myoblasts resulted in an increase of apoptotic cells (Figure 5b, *P *= 0.0095). Immunoblotting revealed that the protein levels of cleaved PARP, caspase‐8, caspase‐3 and pro‐apoptotic protein Bax, cytochrome *c* were greatly upregulated and the level of anti‐apoptotic protein Bcl‐2 was declined with PHGDH knockdown (Figure 5c). To make a further verification of the role of PHGDH in P4HB‐mediated apoptosis, C2C12 myoblasts with PHGDH knockdown were treated with stable P4HB‐depleted YES2‐EVs. As a result, knockdown of PHGDH reversed the inhibitory effect of P4HB depletion on cisplatin‐induced C2C12 myoblast apoptosis (Figure 5d).

However, the underlying mechanisms by which PHGDH regulates apoptosis are not fully understood. Previous study demonstrated that knockdown of PHGDH reduced Bcl‐2 expression in human cervical adenocarcinoma (Jing et al., 2015), but the interaction between PHGDH and Bcl‐2 remains unclear. So, we supposed that PHGDH could interact with Bcl‐2. To ascertain this hypothesis, we performed co‐immunoprecipitation assay of 293T cells transfected with V5‐PHGDH plasmid and found that PHGDH was co‐immunoprecipitated by Bcl‐2 (Figure 5e, top panel). A reciprocal co‐immunoprecipitation assay was then tested with the same transfection as above, and the result further validated the interaction between PHGDH and Bcl‐2 (Figure 5e, bottom panel). Furthermore, we observed that PHGDH was colocalized with Bcl‐2 by immunofluorescence in C2C12 myoblasts (Figure 5f). To investigate the PHGDH regulation of Bcl‐2, we examined the expression levels of Bcl‐2 in C2C12 myoblasts transfected with PHGDH siRNA. Bcl‐2 protein levels, but not the mRNA levels were markedly decreased by PHGDH siRNA (Figure 5g), indicating that PHGDH regulated Bcl‐2 expression through the post‐translational manner. The CHX pulse‐chase assay was performed in PHGDH knockdown C2C12 myoblasts and we found that PHGDH depletion strikingly shorten Bcl‐2 half‐life (Figure 5h. Additionally, an in vitro ubiquitination assay unveiled that the ubiquitination level of Bcl‐2 was greatly increased when PHGDH was knocked down in C2C12 myoblasts (Figure 5i). In conclusion, these findings indicated that depletion of PHGDH downregulated Bcl‐2 expression by increasing its ubiquitination level, and ubiquitin‐proteasome pathway regulated the interaction between PHGDH and Bcl‐2.

### 3.6 The P4HB inhibitor inhibits apoptosis in vitro and in vivo

**FIGURE 6:**
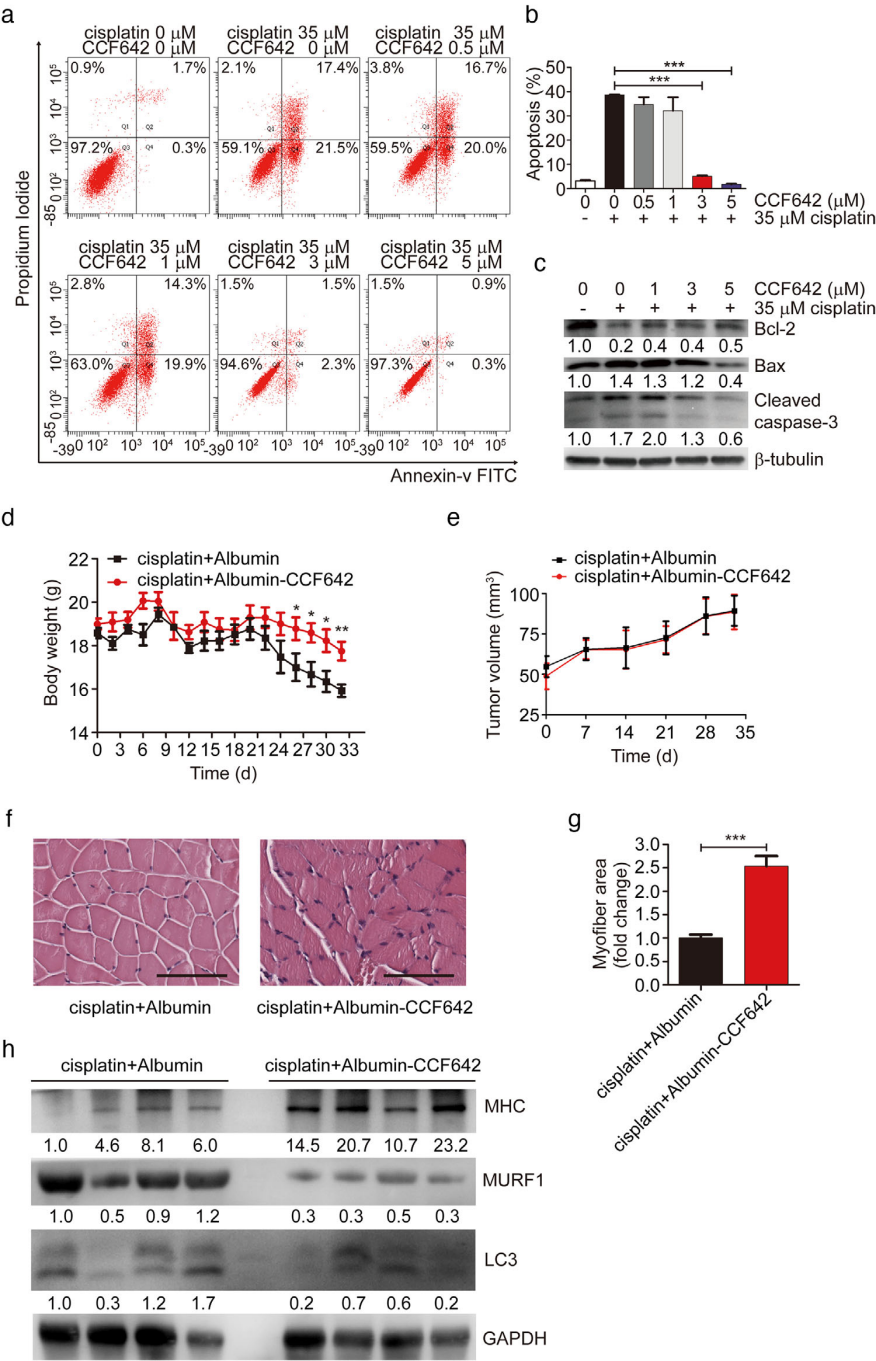
The P4HB inhibitor inhibits apoptosis in vitro and in vivo. (a and b) For apoptosis analysis, C2C12 myoblasts were treated with 0.1% DMSO (vehicle control), or 0 μM, 0.5 μM, 1 μM, 3 μM, and 5 μM CCF642 in combination with 35 μM cisplatin for 24 h. The cells were collected and stained with Annexin V and PI. (c) Western blot analysis of apoptotic markers in C2C12 myoblasts treated with indicated concentrations of CCF642 in combination with 35 μM cisplatin for 24 h. (d) Body weight of YES2‐bearing mice after intraperitoneal injections of albumin vehicle (10 mg/kg, n = 9) or CCF642 (10 mg/kg, n = 9) accompanied with cisplatin (5 mg/kg) three times a week. (e) Tumour volume of YES2‐bearing mice after intraperitoneal injections of albumin vehicle (10 mg/kg, n = 9) or CCF642 (10 mg/kg, n = 9) accompanied with cisplatin (5 mg/kg) three times a week. (f) Representative micrographs of H&E histology of GA muscle in YES2‐bearing mice injected with cisplatin and CCF642 (n = 9), relative to YES2‐bearing mice injected with cisplatin and albumin (n = 9). Scale bars, 100 μm. (g) Quantification of the average myofiber cross‐sectional areas of GA muscle after injections of cisplatin with CCF642 or cisplatin with albumin into YES2‐bearing mice. (h) Western blotting in vivo of MHC, MURF1 and LC3 protein in GA muscle of YES2‐bearing mice injected with cisplatin and CCF642 (n = 4), compared to YES2‐bearing mice injected with cisplatin and albumin (n = 4)

According to the observations above that P4HB could induce apoptosis in vitro and contribute to muscle wasting *in vivo*, we assumed P4HB might serve as a potential therapeutic target for muscle wasting. To examine this supposition in detail, we treated C2C12 myoblasts with CCF642, a novel specific small molecule inhibitor of protein disulfide isomerase (Vatolin et al., 2016). Apoptotic assay results indicated that the percentage of cisplatin‐induced apoptotic cells decreased in a dose‐dependent manner treated with CCF642. After concomitant incubation with 35 μM cisplatin for 24 h, the total apoptotic percentages of apoptotic cells in C2C12 myoblasts treated with 0.1% DMSO (vehicle control) reached 38.67%. Treatment of 3 μM CCF642 significantly reduced apoptotic rates to 5.17%. With the increase of drug concentration to 5 μM, the percentage of apoptotic cells dropped to 1.73% (Figure 6, a and b, *P* < 0.0001). The similar tendency was also observed in L6 rat myoblasts after treatment with CCF642 (Fig. S9, *P *= 0.0013 when CCF642 concentration is 3 μM; *P *= 0.001 when CCF642 concentration is 5 μM). In addition, the levels of apoptosis‐related marker, cleaved caspase‐3, and proapoptotic protein, Bax, were also attenuated in a dose‐dependent manner, whereas antiapoptotic protein, Bcl‐2, was upregulated in the same manner (Figure 6c). These results indicated that CCF642 showed antiapoptotic effects on cisplatin‐induced C2C12 myoblasts. Furthermore, in differentiated C2C12 myotubes induced by cisplatin, treatment with CCF642 increased myotube diameter, elevated expression level of MHC and decreased levels of MURF1 and LC3 (Fig. S10A‐C).

**FIGURE 7:**
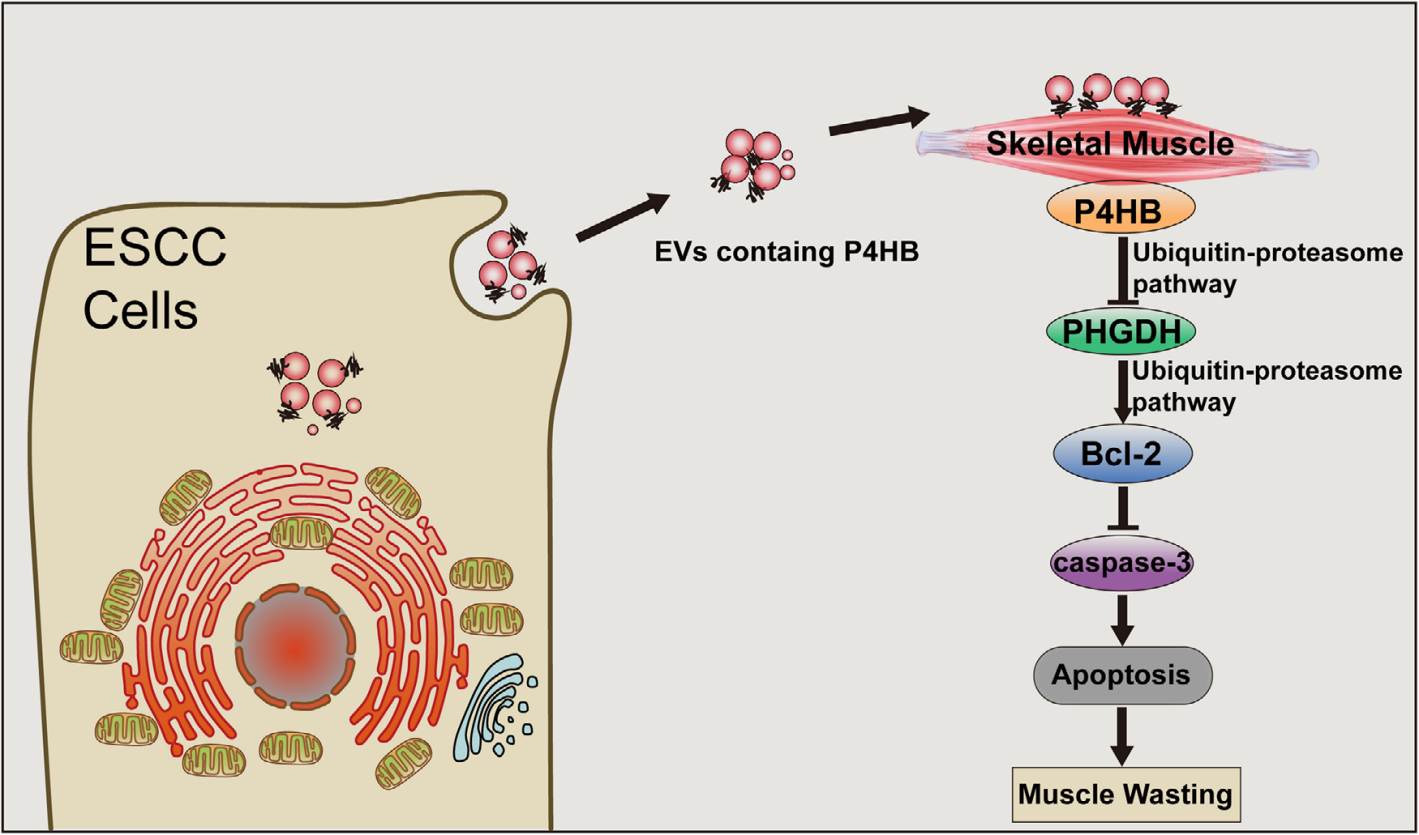
Schematic diagram describing the role of P4HB in the mechanism. Schematic diagram of the mechanism for EVs containing P4HB promoting ESCC‐associated muscle wasting by regulating PHGDH/Bcl‐2/caspase‐3 pathway

Then we used the YES2 model of ESCC‐associated cachexia to test the effect of CCF642 on prevention of muscle wasting *in vivo*. YES2 cells were subcutaneously implanted into nude mice. Ten days later, when tumour volume reached to 50 mm^3^, CCF642 (10 mg/kg) or albumin vehicle concomitant with cisplatin (5 mg/kg) were intraperitoneally (i.p.) administrated into nude mice. The treatment was three times a week for 4 weeks. Administration of CCF642 prevented body weight loss (Figure 6d, *P *= 0.003). However, CCF642 treatment did not result in changes in tumour volume during the experimental period (Figure 6e). CCF642 treatment also prevented cachectic muscle wasting (Figure 6, f and g, *P *= 0.0002), increased MHC protein level and downregulated the protein levels of MURF1 and LC3 in GA muscle as compared to albumin vehicle (Figure 6h). Moreover, adipocyte triglyceride (TG) content in eWAT was higher in response to CCF642 treatment with cisplatin than albumin with cisplatin (Fig. S10D). Histopathological analysis of eWAT revealed that CCF642 treatment prevented adipose tissues wasting (Fig. S10E). In addition, CCF642 treatment could prevent inflammation events (Fig. S10F‐I).

Furthermore, we also compared body weight, GA muscle weight, tumour volume and H&E analysis supplemented with the pair‐fed group and CCF642 treatment alone group. It was proved that CCF642 had no apparent toxicity (Fig. S11).

Taken together, both our in vitro and in vivo results show that P4HB is a potential therapeutic target, and CCF642 is a promising drug candidate for cachexia treatment in ESCC.

## 4 DISCUSSION

Patients with oesophageal cancer, gastric cancer and pancreatic cancer have the highest prevalence of cachexia (Baracos et al., 2018). Thus, understanding mechanisms underlying cancer‐associated cachexia is crucial to develop intervention strategies for cachexia‐management. However, lack of well‐validated animal models obstructs the investigation of ESCC‐induced cachexia, and few studies reported on the mechanism of ESCC‐associated muscle wasting. In the present study, we offered the first insight into ESCC‐induced cachexia in vivo using a human ESCC xenograft mouse model. In this model, mice inoculated with YES2 cells presented significant features of cachexia‐mediated muscle wasting, which provides a valuable resource for future aetiology and drug screening research for ESCC‐induced cachexia.

In the last decade, much attention was given to EVs in cachexia because of its functions in intercellular communication and tumour progression. Several studies have demonstrated microRNAs in EVs played a pivotal role in the development of cancer‐associated muscle wasting (Chitti et al., 2018; Marinho et al., 2017). For instance, miR‐21‐containing EVs promoted muscle wasting by activating TLR7 (He et al., 2014). In contrast, miR‐486 in the EVs decreased the activation ubiquitin pathway and prevented muscle protein loss (Xu et al., 2012). In this study, we identified P4HB from ESCC‐EVs as an important mediator in muscle wasting. Intriguingly, elevated P4HB levels were observed in cachexia‐inducible cell line YES2, but not in other ESCC cell lines and immortalized oesophageal epithelial cell lines. Overexpression of P4HB in non‐cachexia‐inducible cell line, KYSE150, resulted in the development of significant features of cachexia in nude mice. These findings demonstrated that P4HB acted as a crucial mediator of cancer‐associated cachexia. In addition, elevated levels of P4HB in cells and EVs were observed not only in YES2, but also in various types of cachexia‐inducible cancer cell lines originated from both human and mouse, including AGS, BxPC3, and LLC (Fukawa et al., 2016; He et al., 2014; Yang et al., 2019; Zhang et al., 2017). P4HB‐knockdown LLC‐ EVs also inhibited apoptosis of C2C12 myoblasts induced by cisplatin, suggesting that P4HB might be a critical cachectic factor for pan‐cancer types. Moreover, the expression levels of P4HB were upregulated in ESCC cell lines and ESCC tissues compared to adjacent normal tissues, and P4HB overexpression was correlated with poor survival and tumour differentiation of patients with ESCC. Thus, our study paves the way for investigation of P4HB in ESCC progression and cancer cachexia. However, cachexia‐inducible pancreatic adenocarcinoma cell line AsPC1 (Yang et al., 2019) did not express P4HB. This suggested there were other mechanisms in cancer‐associated cachexia. Moreover, we cannot exclude that other factors also play a role in the pathogenesis of cachexia. In the future, it is necessary to screen other molecules by high‐throughput methods.

Importantly, using our ESCC model of cachexia, we successfully identified PHGDH as a target of P4HB in cachexia‐associated muscle wasting. P4HB directly interacted with PHGDH and downregulated PHGDH stability by increasing ubiquitination level of PHGDH. Knockdown of PHGDH expression in C2C12 greatly attenuated the inhibitory effect of P4HB depletion on apoptosis rate. In addition, we also found Bcl‐2 downregulation in PHGDH‐depletion induced apoptosis in C2C12 myoblasts. We confirmed PHGDH was able to bind to Bcl‐2 and PHGDH knockdown increased the ubiquitination level of Bcl‐2 to accelerate the degradation process and subsequently upregulated the level of cleaved caspase‐3. On the basis of these findings, we proposed that ESCC‐EVs with high levels of P4HB evoked muscle wasting by activating the PHGDH/Bcl‐2/caspase‐3 pathway.

Cancer cachexia always correlates with aggravated toxicity and complications of cancer therapy. Some of clinical trials have provided therapeutically useful approaches to prevent body weight loss and increase muscle power. However, none of them changes the tolerance of cachexia patients in chemotherapy toxicity (Bourdel‐Marchasson et al., 2014), and no drug has been approved to relieve this circumstance. Employing in vitro and in vivo ESCC‐induced cachexia model, we found that protein disulfide isomerase inhibitor, CCF642, prevented cisplatin‐induced apoptosis of myoblasts, myotube atrophy and ameliorated cachectic features *in vivo*, including muscle and adipose tissues wasting and inflammation. These results suggested that CCF642, proved to have no apparent toxicity, might have promising potential for clinical intervention of ESCC‐induced cachexia, and relieve the adverse effects of chemotherapy. We acknowledge that the study remains underpowered to detect the long‐term cytotoxicity *in vivo* and devoid of adequate cases for prospective clinical trials. It's urgently mandatory for basic research and clinical trial collaboration to realize the idea ‘from bench to beside’.

Collectively, our study provides a novel mouse model of ESCC‐induced cachexia, unveils the molecular mechanism and a small‐molecule inhibitor of ESCC‐induced muscle wasting and cachexia, foretelling the feasibility of overturning this devastating adverse effect. It is noteworthy to guide a therapeutic direction for potential anti‐cachexia treatment in ESCC.

## CONFLICTS OF INTEREST

No potential conflicts of interest were disclosed.

## AUTHOR CONTRIBUTIONS

Conception and design: X. Gao, Y. Wang, Qimin Zhan. Development of methodology: X. Gao, Y. Wang, Qimin Zhan. Acquisition of data (provided animals and provided facilities, etc.): X. Gao, Y. Wang, X. Chen, L. Zheng, D. Yang. Analysis and interpretation of data (e.g., statistical analysis, biostatistics, computational analysis): X. Gao, Y. Wang, G. Wang. Writing, review, revision, and/or correction of the manuscript: X. Gao, Y. Wang, F. Lu, Y. Cao, W. Zhang, J. Chen, Qimin Zhan. Administrative, technical, or material support (i.e., reporting or organizing data, constructing databases): X. Gao, Y. Wang, M. Fu, L. Ma. Study supervision: Y. Song, Qimin Zhan.

## Supporting information

Supporting Information
