# Supplementary material for: Extracellular vesicles derived from oesophageal cancer containing P4HB promote muscle wasting via regulating PHGDH/Bcl‐2/caspase‐3 pathway
Source: J Extracell Vesicles. 2021 Mar 10;10(5):e12060. doi: 10.1002/jev2.12060 (PMC7944388; doi:10.1002/jev2.12060)
Supplement: Supplementary file 1 — Supporting Information [file JEV2-10-e12060-s001.docx]

**Supplementary Materials**

**
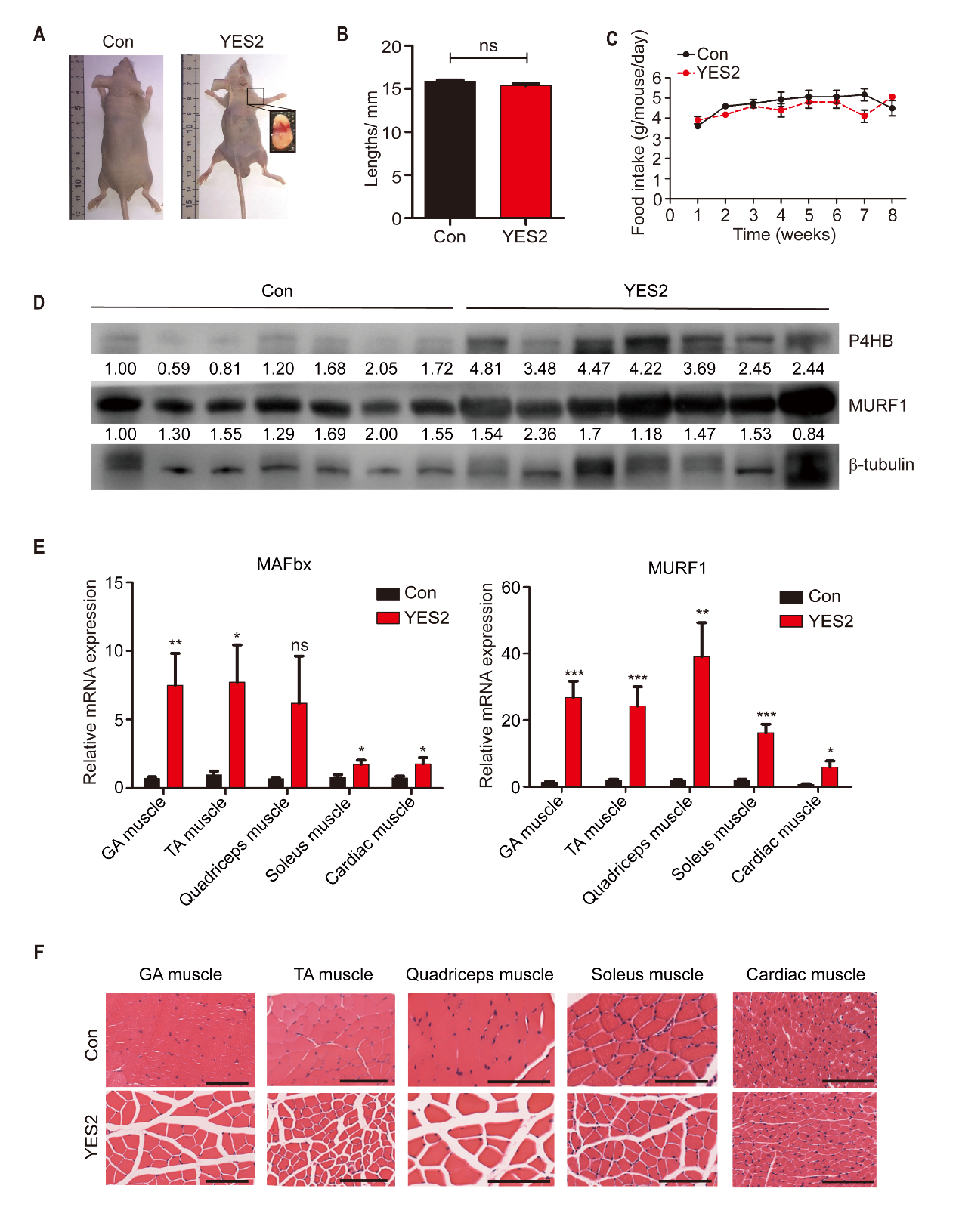
**

Fig. S1. YES2-bearing mice present apparent muscle wasting. (**A**) Mouse images of xenograft-bearing Balb/c nude mice subcutaneously implanted with YES2 cells and of respective non-tumor-bearing control (Con) mice on the left in Figure 1C. The right panel showed amplified xenograft tumor image. (**B**) Analysis of foot lengths of mice bearing YES2 cells (*n*=5) compared to respective non-tumor-bearing control (Con; *n*=5) mice. (**C**) Average daily food intake of mice bearing YES2 cells (*n*=5) compared to respective non-tumor-bearing control (Con; *n*=5) mice for 8 weeks. (**D**) Western blot of P4HB and MURF1 protein in GA muscle from non-tumor-bearing control mice (*n*=7) and YES2-bearing mice (*n*=7). (**E**) The mRNA expression of muscle atrophy markers, *MAFbx* and *MURF1* in GA muscles, TA muscles, quadriceps muscles, soleus muscles and cardiac muscles in non-tumor-bearing mice (*n* = 9) and YES2-bearing mice (*n* = 9). (**F**) Representative micrographs of H&E histology of GA muscles, TA muscles, quadriceps muscles, soleus muscles and cardiac muscles in non-tumor-bearing mice (*n* = 9) and YES2-bearing mice (*n* = 9). Scale bars, 100 μm.


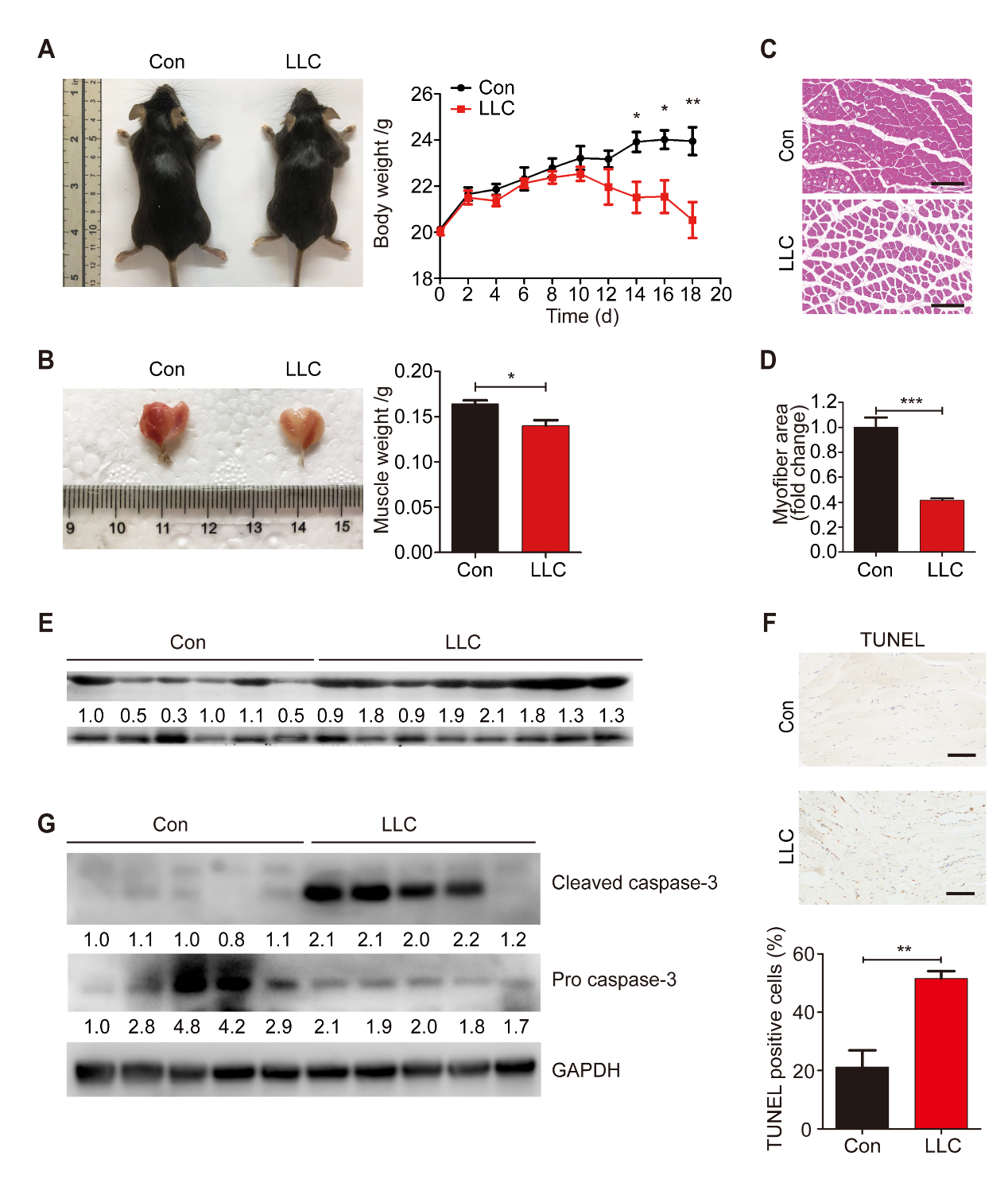


Fig. S2. Modeling cancer-induced cachexia with LLC cells. (**A**) Mouse images (left) and body weight changes (right) of tumor-bearing C57BL/6J mice subcutaneously implanted with LLC cells (*n*=8) and of respective non-tumor-bearing control (Con; *n*=5) mice. Mouse images were taken at 18 days after LLC injection. (**B**) GA muscle images (left) and muscle weight analysis in LLC-bearing mice (*n*=8) and non-tumor-bearing control (Con; *n*=5) mice. (**C**) Representative micrographs of H&E histology of GA muscle in LLC-bearing mice (*n*=8), relative to non-tumor-bearing control mice (*n*=5). Scale bars, 150 μm. (**D**) Quantification of the myofiber cross-sectional areas in LLC-bearing mice versus non-bearing mice. (**E**) Western blot analysis of MURF1 protein in GA muscle from non-tumor-bearing control mice (*n*=6) and LLC-bearing mice (*n*=8). (**F**) TUNEL assay of GA muscle in LLC-bearing mice (*n*=8), relative to non-tumor-bearing control mice (*n*=5) and quantification of percentage of TUNEL positive cells. Scale bars, 100 μm. (**G**) Western blot analysis of cleaved caspase-3 protein and pro caspase-3 protein in GA muscle from non-tumor-bearing control mice (*n*=5) and LLC-bearing mice (*n*=5).


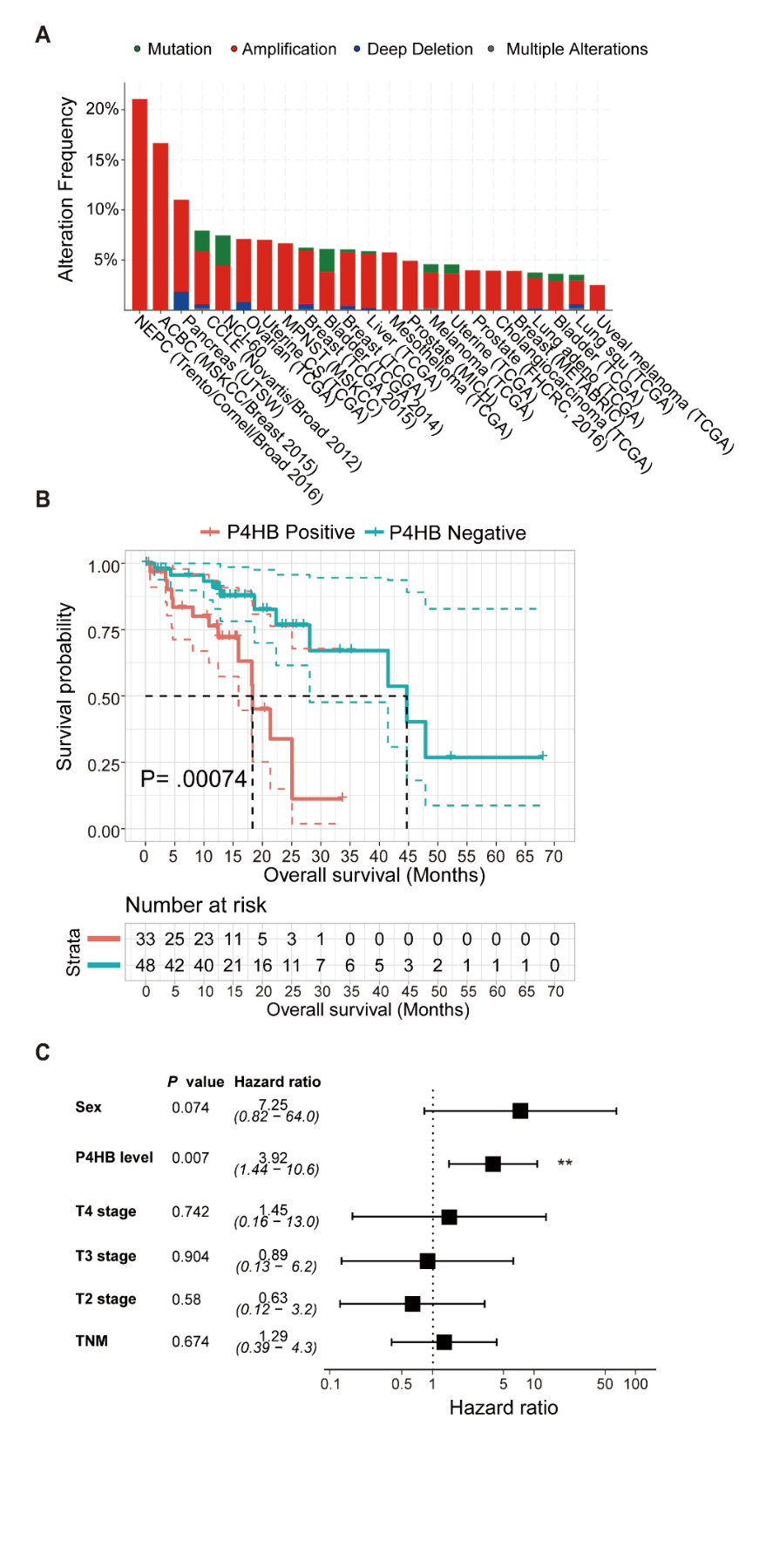


Fig. S3. The expression level of P4HB could be an independent prognosis factor for ESCC (**A**) The frequency of P4HB somatic alterations across multiple cancers. (**B**) Kaplan–Meier survival analysis of patients with ESCC stratified by P4HB expression level shows poor overall survival utilizing published TCGA transcriptome dataset. (**C**) Multivariate Cox regression survival analysis of associations between survival and various clinicopathologic factors (Sex, P4HB level, tumor stages, lymph node metastasis (TNM)) with aforementioned dataset. HRs and 95% CIs are plotted for each variable.

**
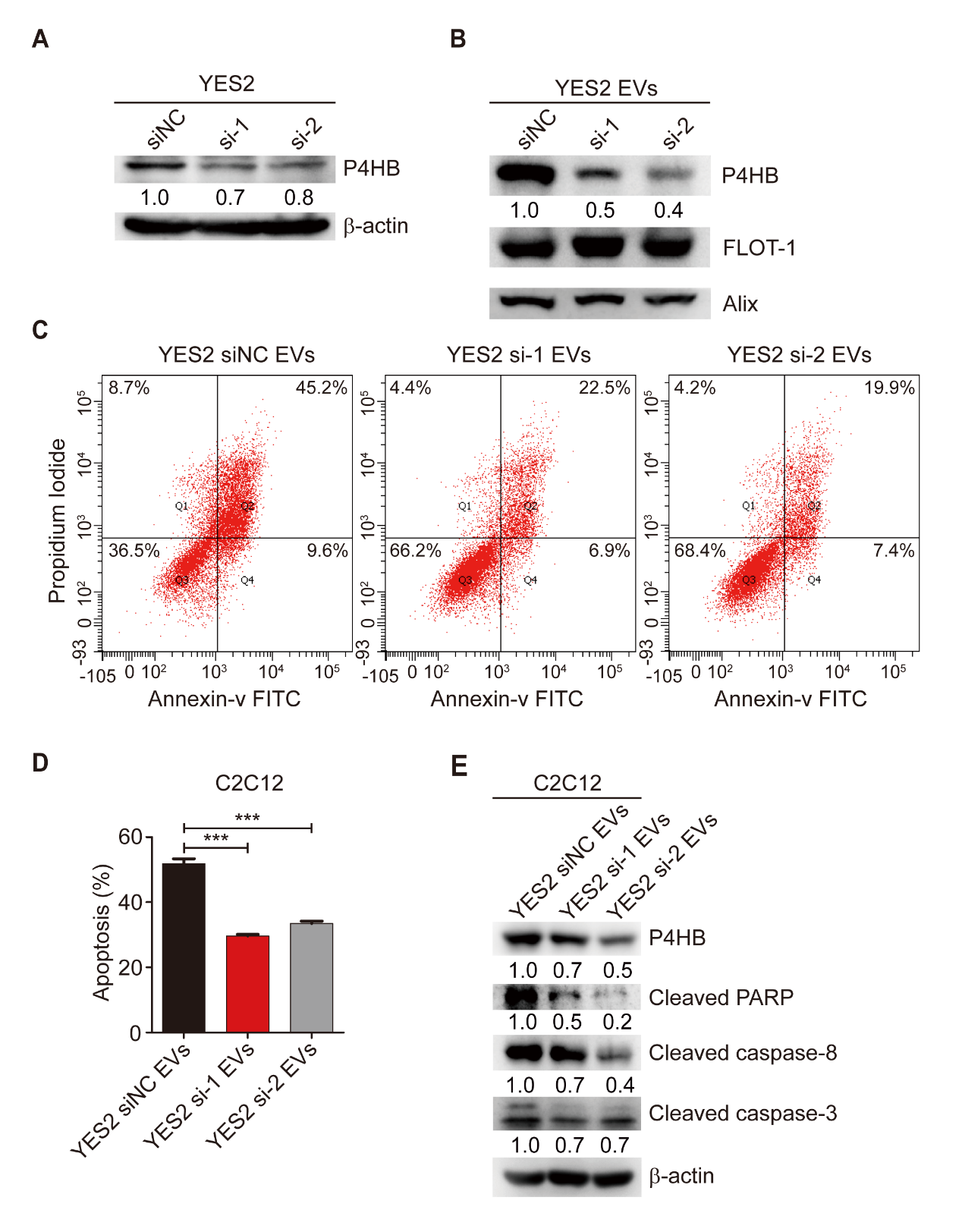
**

Fig. S4. The EVs with P4HB depletion inhibit apoptosis in C2C12 myoblasts. (**A**) Depleted P4HB levels after transfecting siRNA in YES2 cell lines were measured by Western blot. (**B**) The P4HB levels in EVs secreted by YES2 transfected with P4HB siRNA. (**C** and **D**) For apoptosis analysis, C2C12 myoblasts were treated with EVs (10 μg) for 24 h derived from YES2 cells transfected with P4HB siRNA induced by 35 μM cisplatin. (**E**) Western blot analysis of apoptotic markers in C2C12 myoblasts treated with EVs (10 μg) for 24 h derived from YES2 cells transfected with P4HB siRNA induced by 35 μM cisplatin.


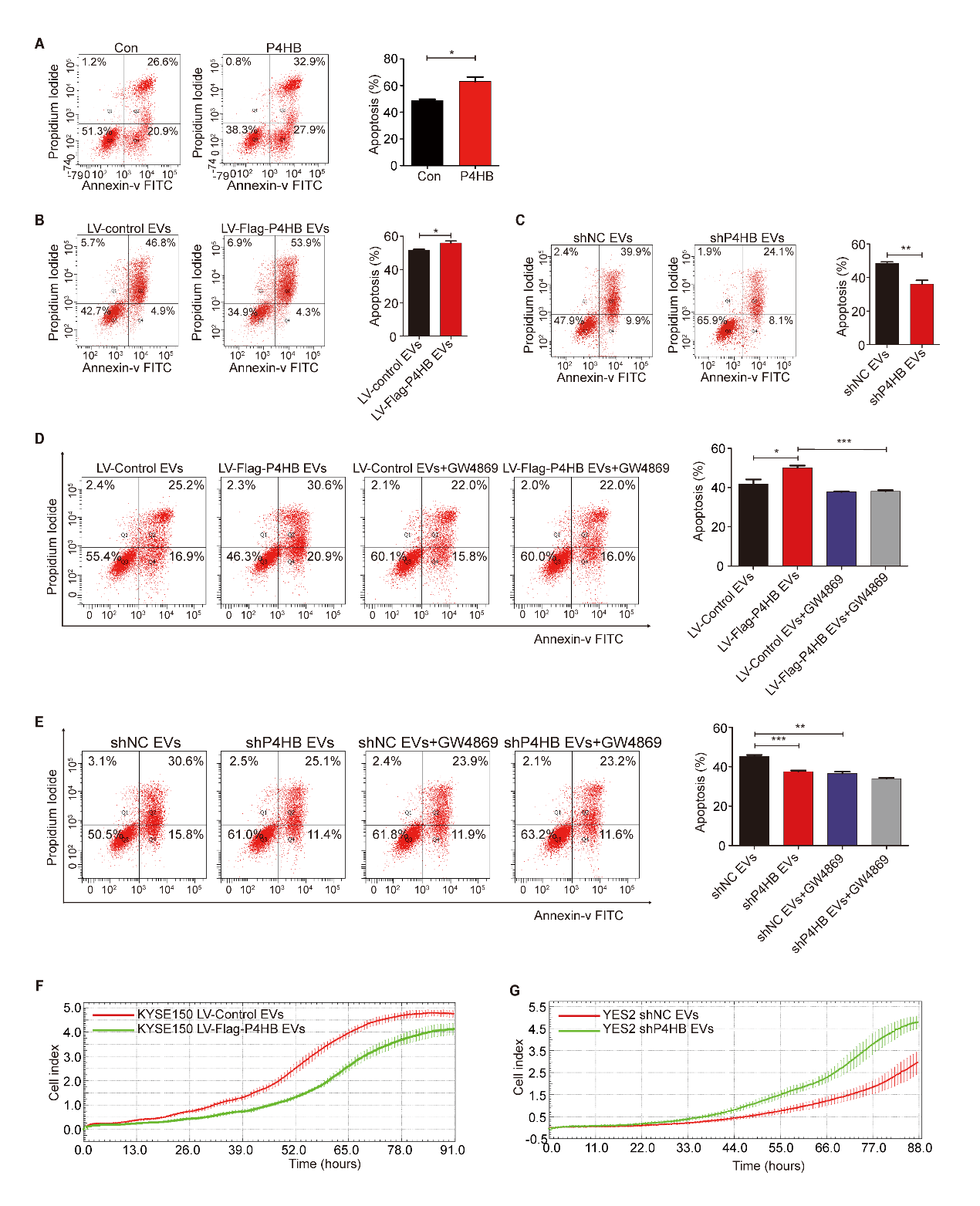


Fig. S5. Mimicking artificial EVs packaging P4HB results in enhanced apoptosis in C2C12 myoblasts, and also stable P4HB overexpression induces apoptosis and inhibits cell proliferation in C2C12 myoblasts. (**A**) The apoptosis assay in C2C12 myoblasts transfected with P4HB plasmid induced by 35 μM cisplatin for 24h. (**B** and **C**) For apoptosis analysis, C2C12 myoblasts were treated with EVs (10 μg) derived from KYSE150 cells with stable overexpressed P4HB and YES2 cells with stable depleted P4HB in combination with 35 μM cisplatin for 24 h. (**D**) The assay of apoptosis in C2C12 myoblasts treated with KYSE150 EVs (LV-control and stable P4HB-overexpressed) and nSMase, GW4869 induced by 35 μM cisplatin for 24 h. (**E**) The assay of apoptosis in C2C12 myoblasts treated with YES2 EVs (shNC and stable P4HB-depleted) and GW4869 induced by 35 μM cisplatin for 24 h. (**F** and **G**) The cell growth curve of C2C12 myoblasts treated with EVs released from KYSE150 cells with stable overexpressed P4HB or YES2 cells with stable depleted P4HB.


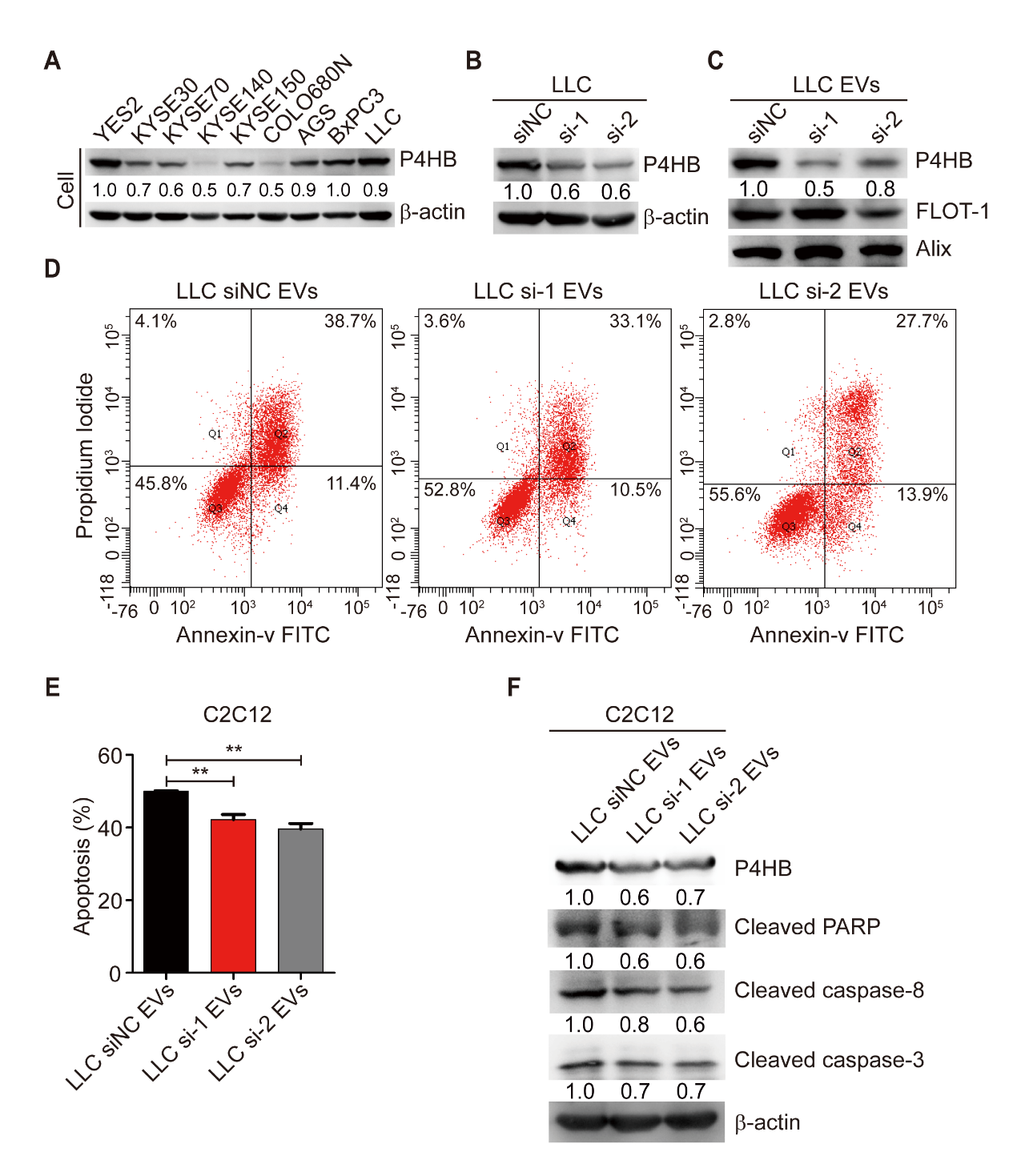


Fig. S6. P4HB is upregulated in cachexia-induced cancer cells and elevated P4HB in EVs is crucial to LLC cells-induced apoptosis in C2C12 myoblasts. (**A**)The P4HB levels in ESCC cell lines and other types of cancer cells known as cachexia-induced cancer cells (AGS, BxPC3, LLC) were analyzed by immunoblotting. (**B**) Depleted P4HB levels after transfecting siRNA in LLC cell lines were measured by western blot. (**C**) The P4HB levels in EVs secreted by LLC transfected with P4HB siRNA. (**D** and **E**) For apoptosis analysis, C2C12 myoblasts were treated with EVs (10 μg) for 24 h derived from LLC cells transfected with P4HB siRNA induced by 35 μM cisplatin. (**F**) Western blot analysis of apoptotic markers in C2C12 myoblasts treated with EVs (10 μg) for 24 h derived from LLC cells transfected with P4HB siRNA induced by 35 μM cisplatin.

**
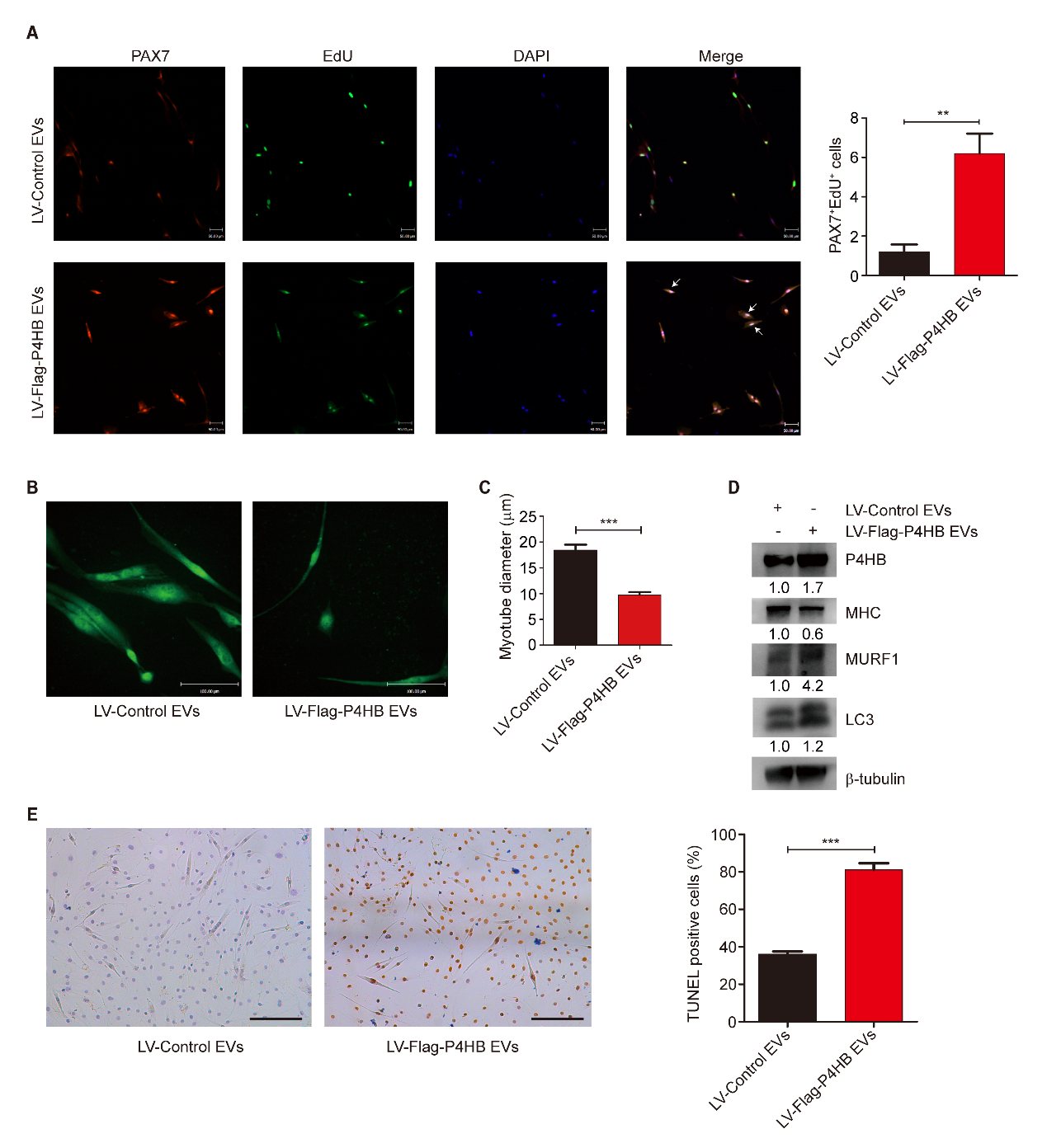
**

Fig. S7. EVs containing P4HB induces activation of satellite cells, muscle atrophy and autophagy of C2C12 myotubes *in vitro*. (**A**) Satellite cells isolated from mice were incubated with EVs from KYSE150 cells with stable P4HB overexpression for 24h. Cells were stained with PAX7 and EdU. Nuclei were counterstained with DAPI. Scale bars, 50 μm. (**B, C**) Immunofluorescent staining for C2C12 myotubes and quantification for myotube diameter after treated with LV-Flag-P4HB EVs and 35 μM cisplatin for 24 h (*n*= 7 per group, scale bars, 100 μm); (**D**) Western blotting *in vitro* of MHC, MURF1 and LC3 protein in C2C12 myotubes treated with LV-Flag-P4HB EVs and 35 μM cisplatin for 24 h. (**E**) TUNEL assay for C2C12 myotubes after treated with LV-Flag-P4HB EVs and 35 μM cisplatin for 24 h and quantification of percentage of TUNEL positive myotubes (*n* =3 per group; scale bars, 30 μm).

**
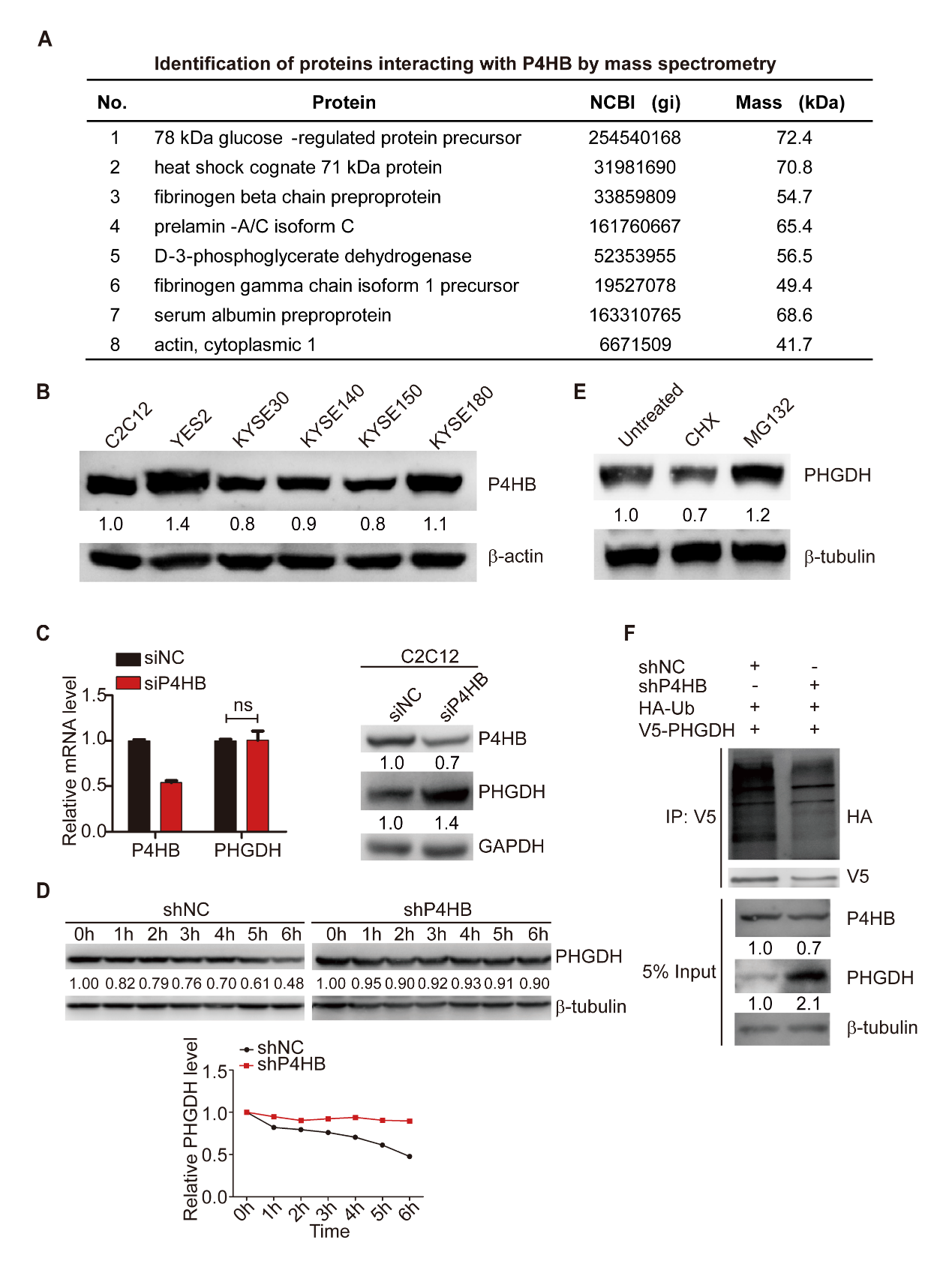
**

Fig. S8. P4HB knockdown deubiquitinates PHGDH and inhibits its degradation. (**A**) Identification of proteins interacting with P4HB by mass spectrometry. (**B**) The P4HB levels in C2C12 myoblast, YES2 cell line and other ESCC cell lines were analyzed by immunoblotting. (**C**)The mRNA levels and protein levels of P4HB and PHGDH were analyzed by RT-qPCR assay (left) and immunoblotting assay (right) in C2C12 myoblasts with depleted P4HB. (**D**) C2C12 myoblasts with stable depleted P4HB were treated with 100 μg/ml CHX and harvested at the indicated time point. The cell lysates were subjected to immunoblotting and PHGDH expression was quantified by ImageJ software. (**E**) The protein levels of PHGDH in C2C12 myoblasts in normal conditions, with 100 μg/ml CHX and 10 μM MG132 for 8h, respectively were analyzed by immunoblotting. (**F**) C2C12 myoblasts with stable depleted P4HB were co-transfected with HA-Ub and V5-PHGDH plasmid for 36h and then cells were incubated with 10 μM MG132 for 6 h. PHGDH ubiquitination level was assessed by immunoprecipitation.


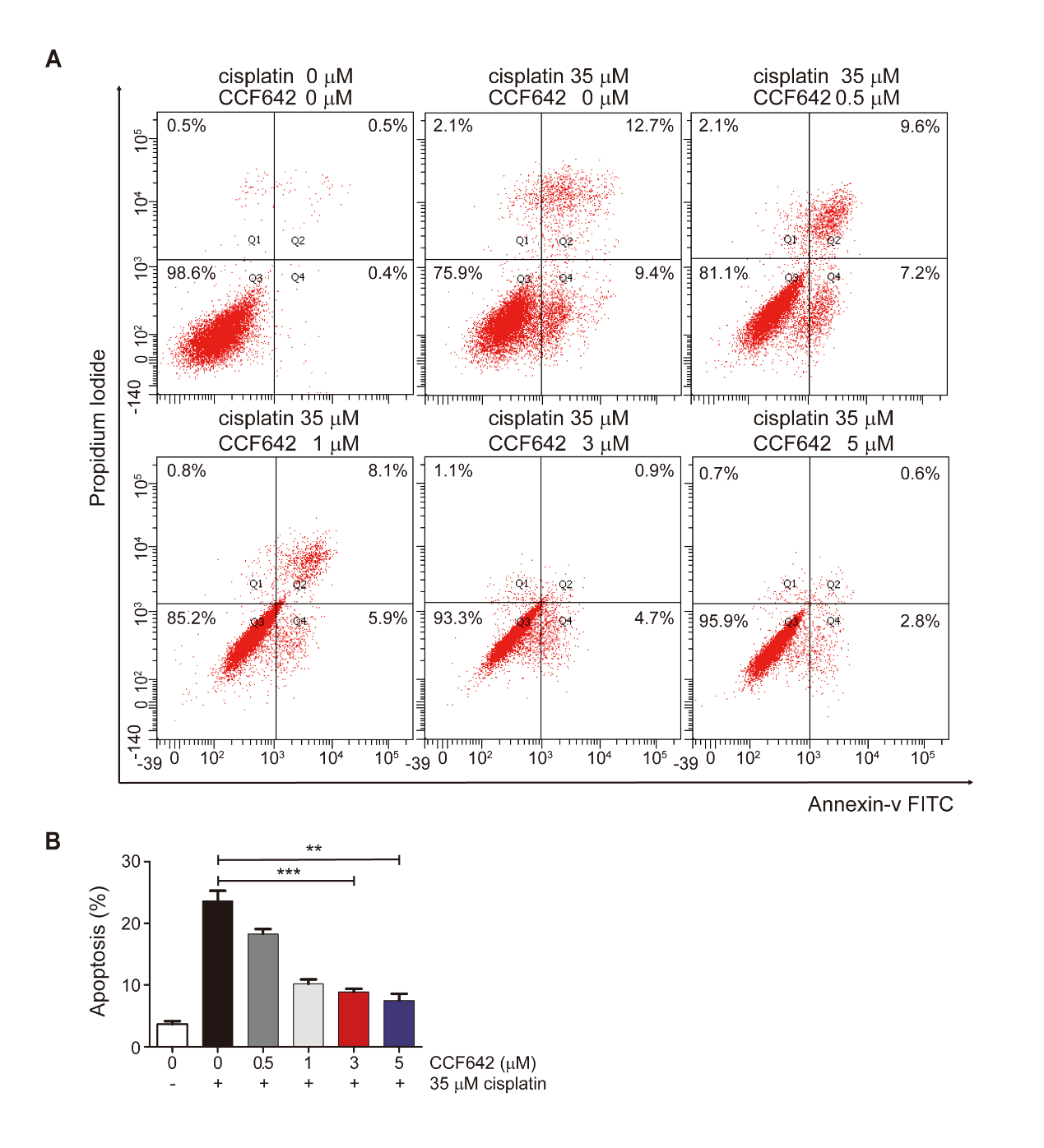


Fig. S9. The P4HB inhibitor (CCF642) inhibits apoptosis in L6 rat myoblasts. (**A** and **B**) For apoptosis analysis, L6 myoblasts were treated with 0.1% DMSO (vehicle control), or 0 μM,0.5 μM, 1 μM, 3 μM and 5 μM CCF642 in combination with 35 μM cisplatin for 24 h. The cells were collected and stained with annexin V and PI.


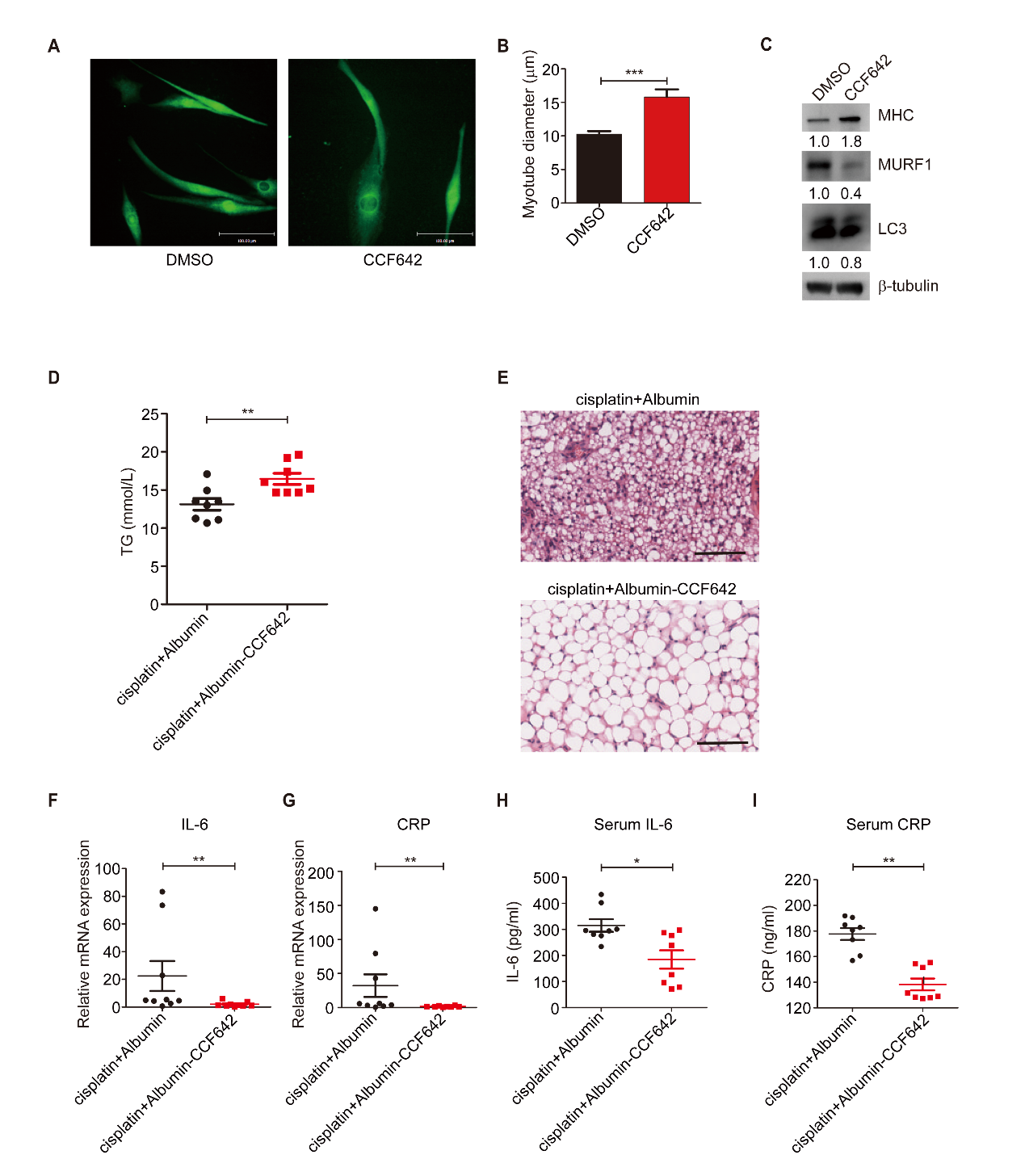


Fig. S10. The P4HB inhibitor (CCF642) reverses cachexia phenotype, including muscle atrophy *in* *vitro*, fat metabolism and inflammation *in vivo*. (**A** and **B**) Immunofluorescent staining for C2C12 myotubes and quantification for myotube diameter after treated with 3 μM CCF642 and 35 μM cisplatin in C2C12 myotubes for 24 h (*n*= 7 per group, scale bars, 100 μm). (**C**) Western blotting *in vitro* of MHC, MURF1 and LC3 levels in C2C12 myotubes treated with 3 μM CCF642 and 35 μM cisplatin for 24h. (**D**) Quantification of eWAT TG levels of YES2-bearing mice injected with cisplatin and CCF642 (*n* = 8), relative to YES2-bearing mice injected with cisplatin and albumin (*n* = 8); (**E**) Representative micrographs of H&E histology of eWAT in YES2-bearing mice injected with cisplatin and CCF642 (*n* = 8), relative to YES2-bearing mice injected with cisplatin and albumin (*n* = 8). Scale bars, 100 μm. (**F**) The mRNA expression of inflammatory marker *IL-6* in GA muscle of YES2-bearing mice injected with cisplatin and CCF642 (*n* = 8), relative to YES2-bearing mice injected with cisplatin and albumin (*n* = 8). (**G**) The mRNA expression of inflammatory marker *CRP* in GA muscle of YES2-bearing mice injected with cisplatin and CCF642 (*n* = 8), relative to YES2-bearing mice injected with cisplatin and albumin (*n* = 8). (**H**) Serum concentrations of inflammatory marker IL-6 in YES2-bearing mice injected with cisplatin and CCF642 (*n* = 8), relative to YES2-bearing mice injected with cisplatin and albumin (*n* = 8). (**I**) Serum concentrations of inflammatory marker CRP in YES2-bearing mice injected with cisplatin and CCF642 (*n* = 8), relative to YES2-bearing mice injected with cisplatin and albumin (*n* = 8).


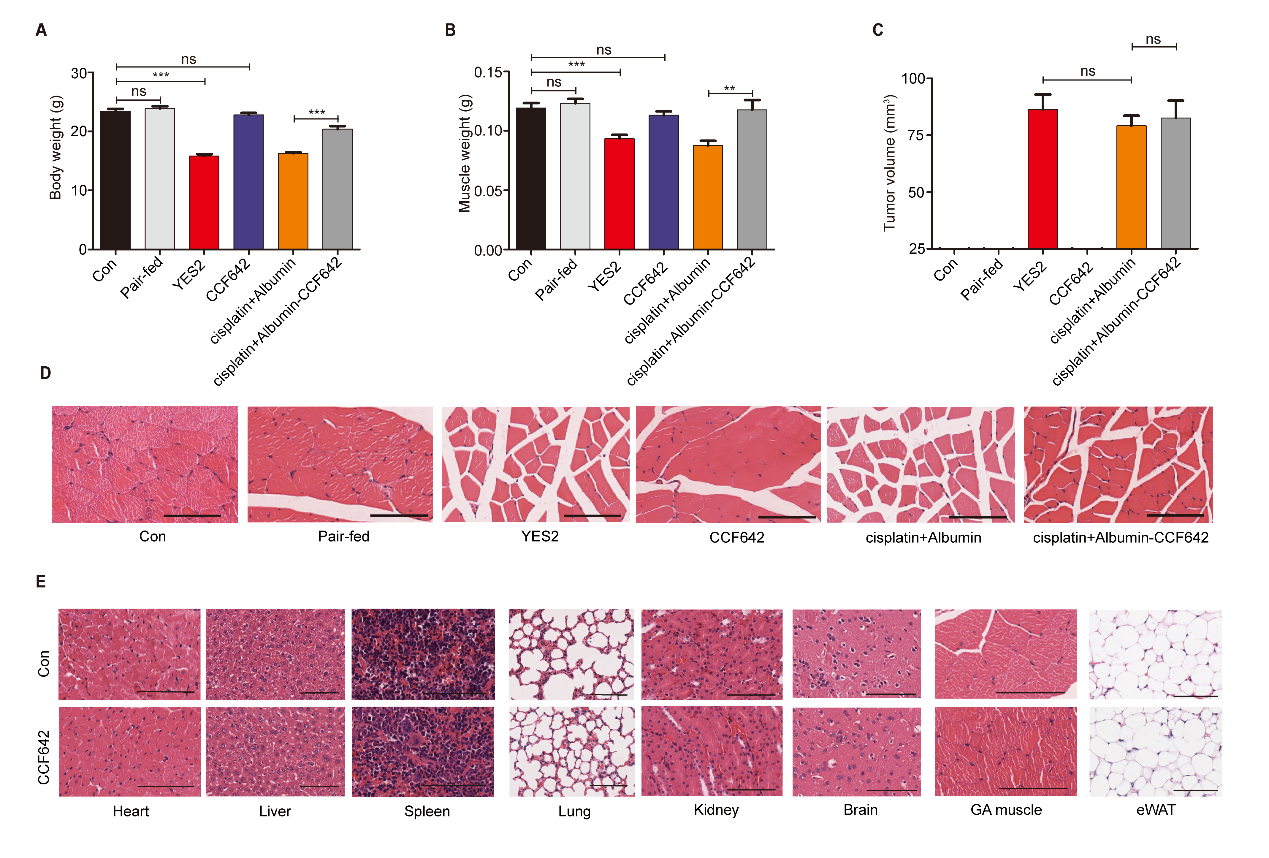


Fig. S11. The P4HB inhibitor (CCF642) prevents muscle wasting with no apparent toxicity. (**A**) Body weight analysis of the group of treatment naive (mice with no cancer cells implantation and no drug treatment, *n*=10), pair-fed treatment group (mice with the same amount of food as YES2 alone group, *n*=10), YES2 group (xenograft-bearing Balb/c nude mice subcutaneously implanted with YES2, *n*=9), the group of CCF642 administration alone (mice administrated with CCF642 and with no cancer cells implantation, *n*=10), cisplatin and Albumin (YES2-bearing mice after intraperitoneal injections of albumin vehicle accompanied with cisplatin, *n* = 8), cisplatin and CCF642 (YES2-bearing mice after intraperitoneal injections of CCF642 accompanied with cisplatin, *n* = 8). (**B**) GA muscle weight analysis of the above corresponding groups. (**C**) Tumor volume analysis of the group of treatment naive (mice with no cancer cells implantation and no drug treatment, *n*=10), pair-fed treatment group (mice with the same amount of food as YES2 alone group, *n*=10), YES2 group (xenograft-bearing Balb/c nude mice subcutaneously implanted with YES2, *n*=9), the group of CCF642 administration alone (mice administrated with CCF642 and with no cancer cells implantation, *n*=10), cisplatin and Albumin (YES2-bearing mice after intraperitoneal injections of albumin vehicle accompanied with cisplatin, *n* = 8), cisplatin and CCF642 (YES2-bearing mice after intraperitoneal injections of CCF642 accompanied with cisplatin, *n* = 8). (**D**) Representative micrographs of H&E histology of GA muscle of the above corresponding groups. Scale bars, 100 μm. (**E**) Representative micrographs of H&E histology of heart, liver, spleen, lung, kidney, brain, GA muscle and eWAT in mice administrated with CCF642 (*n* = 10), relative to healthy mice (*n* = 10). Scale bars, 100 μm.

**Supplementary Table 1**

**Table S1. Association of P4HB expression with clinicopathological features of 103 ESCC samples**

| **Clinicopathologic features** | **Total cases** | **P4HB Expression** | |  |
| --- | --- | --- | --- | --- |
|  | **(%)** | **Positive (%)** | **Negative (%)** | ***P*** |
| Gender |  |  |  |  |
| Male | 85(82.5) | 48(56.5) | 37(43.5) | 0.251 |
| Female | 18(17.5) | 8(44.4) | 10(55.6) |  |
| Age |  |  |  |  |
| ≥60 | 79(76.7) | 40(50.6) | 39(49.4) | 0.125 |
| <60 | 24(23.3) | 16(66.7) | 8(33.3) |  |
| Differentiation |  |  |  |  |
| High | 42(40.8) | 17(40.5) | 25(59.5) | **0.016** |
| Low/moderate | 61(59.2) | 39(63.9) | 22(36.1) |  |
| T_Stage |  |  |  |  |
| T1/T2 | 26(25.2) | 12(46.2) | 14(53.8) | 0.228 |
| T3 | 77(74.8) | 44(57.1) | 33(42.9) |  |
| Overall Survival |  |  |  |  |
| Dead | 64(62.1) | 37(57.8) | 27(42.4) | 0.243 |
| Alive | 39(37.9) | 19(48.7) | 20(51.3) |  |

**Supplementary Table 2**

**Table S2. The clinicopathological characteristics of 103 ESCC samples**

| **ID** | **Gender** | **Age** | **T stage** | **Differentiation** | **Overall Survival** | **Time (Month)** | **Matched adjacent tissues** |
| --- | --- | --- | --- | --- | --- | --- | --- |
| D08A0838 | Male | 69 | T3 | High | Dead | 20 | YES |
| D08A0841 | Male | 80 | T3 | Low | Dead | 10 | YES |
| D08A0843 | Male | 52 | T2 | High | Alive | 78 | YES |
| D08A0878 | Male | 57 | T3 | High | Alive | 77 | YES |
| D08A0887 | Male | 77 | T3 | Low | Dead | 17 | YES |
| D08A0907 | Male | 65 | T3 | Low | Dead | 8 | YES |
| D08A0911 | Male | 76 | T2 | High | Alive | 75 | YES |
| D08A0952 | Female | 58 | T3 | High | Alive | 75 | YES |
| D08A0953 | Male | 60 | T3 | Moderate | Dead | 2 | YES |
| D08A0954 | Male | 80 | T3 | High | Dead | 8 | YES |
| D08A0955 | Male | 57 | T3 | Moderate | Alive | 75 | YES |
| D08A0980 | Male | 72 | T3 | Moderate | Dead | 28 | YES |
| D08A0981 | Male | 64 | T3 | Moderate | Dead | 6 | YES |
| D08A1031 | Male | 60 | T3 | Moderate | Dead | 27 | YES |
| D08A1032 | Male | 64 | T3 | High | Alive | Lost to follow-up | YES |
| D08A1035 | Male | 61 | T2 | Moderate | Dead | 62 | YES |
| D08A1038 | Male | 61 | T3 | High | Dead | 20 | YES |
| D08A1088 | Female | 63 | T2 | Low | Alive | 72 | YES |
| D08A1089 | Male | 65 | T2 | High | Dead | 25 | YES |
| D08A1090 | Male | 56 | T3 | High | Dead | 38 | YES |
| D08A1091 | Male | 81 | T3 | Moderate | Dead | 51 | YES |
| D08A1093 | Male | 49 | T3 | Moderate | Dead | 3 | YES |
| D08A1094 | Female | 68 | T2 | High | Alive | 72 | YES |
| D08A1096 | Male | 52 | T2 | High | Alive | 72 | YES |
| D08A1124 | Male | 66 | T3 | Moderate | Dead | 14 | YES |
| D08A1125 | Male | 65 | T3 | Moderate | Alive | 71 | YES |
| D08A1127 | Female | 81 | T1b | High | Alive | 71 | YES |
| D08A1162 | Female | 67 | T3 | High | Dead | 16 | YES |
| D08A1166 | Male | 71 | T3 | Moderate | Dead | 2 | YES |
| D08A1222 | Male | 55 | T3 | Low | Dead | 15 | YES |
| D08A1224 | Female | 63 | T1b | High | Dead | 0.13 | YES |
| D08A1225 | Male | 73 | T2 | High | Alive | 69 | YES |
| D08A1265 | Male | 67 | T2 | High | Dead | 21 | YES |
| D08A1266 | Male | 63 | T2 | Moderate | Dead | 9 | YES |
| D08A1267 | Male | 66 | T3 | Moderate | Alive | 68 | YES |
| D08A1269 | Male | 51 | T3 | High | Dead | 33 | YES |
| D08A1313 | Male | 69 | T2 | Moderate | Dead | 12 | YES |
| D08A1314 | Male | 67 | T3 | Low | Alive | Lost to follow-up | YES |
| D08A1347 | Female | 72 | T3 | High | Alive | 66 | YES |
| D08A1348 | Male | 72 | T3 | High | Dead | 11 | YES |
| D08A1343 | Male | 63 | T3 | Moderate | Dead | 42 | YES |
| D08A1412 | Male | 59 | T3 | Low | Dead | 3 | YES |
| D08A1416 | Male | 57 | T3 | High | Dead | 8 | YES |
| D08A1459 | Male | 69 | T1 | High | Alive | 63 | YES |
| D08A1460 | Male | 62 | T3 | Moderate | Alive | 63 | YES |
| D08A1461 | Female | 79 | T2 | Low | Dead | 25 | YES |
| D08A1462 | Female | 65 | T3 | Moderate | Dead | 11 | YES |
| D08A1463 | Male | 83 | T3 | High | Dead | 11 | YES |
| D08A1483 | Male | 62 | T3 | High | Dead | 16 | YES |
| D08A1484 | Male | 69 | T1 | Moderate | Alive | 62 | YES |
| D08A1485 | Male | 63 | T3 | Moderate | Alive | 62 | YES |
| D08A1486 | Male | 72 | T3 | Moderate | Dead | 30 | YES |
| D08A1499 | Male | 77 | T3 | Moderate | Dead | 8 | YES |
| D08A1500 | Male | 65 | T2 | Low | Dead | 2 | YES |
| D08A1501 | Male | 75 | T3 | High | Dead | 4 | YES |
| D08A1503 | Male | 69 | T3 | High | Dead | 12 | YES |
| D08A1675 | Male | 76 | T3 | Moderate | Dead | 18 | YES |
| D08A1676 | Male | 73 | T3 | Low | Dead | 16 | YES |
| D08A1680 | Female | 73 | T2 | Low | Dead | 9 | YES |
| D08A1681 | Female | 62 | T3 | Moderate | Dead | 6 | YES |
| D08A1899 | Male | 51 | T3 | Moderate | Alive | 60 | YES |
| D08A1902 | Female | 69 | T3 | Moderate | Alive | 59 | YES |
| D08A1903 | Male | 63 | T2 | Moderate | Alive | Lost to follow-up | YES |
| D08A1904 | Male | 50 | T3 | High | Alive | 59 | YES |
| D08A1909 | Male | 50 | T3 | Low | Dead | 16 | YES |
| D08A1915 | Male | 56 | T3 | Moderate | Alive | Lost to follow-up | YES |
| D08A1916 | Male | 64 | T3 | High | Dead | 15 | YES |
| D08A1924 | Male | 56 | T2 | Moderate | Alive | Lost to follow-up | YES |
| D08A1926 | Male | 59 | T3 | Moderate | Dead | 10 | YES |
| D08A1958 | Male | 63 | T3 | Low | Alive | 56 | YES |
| D08A1959 | Male | 52 | T3 | High | Alive | 56 | YES |
| D08A1960 | Male | 58 | T3 | Moderate | Dead | 10 | YES |
| D08A1965 | Female | 75 | T2 | Moderate | Alive | 55 | YES |
| D08A1972 | Male | 65 | T3 | Low | Alive | Lost to follow-up | YES |
| D08A1973 | Male | 61 | T3 | Moderate | Dead | 1 | YES |
| D08A1975 | Male | 66 | T4b | High | Dead | 1 | YES |
| D08A1977 | Male | 78 | T3 | High | Alive | 55 | YES |
| D08A0880 | Male | 57 | T3 | Moderate | Dead | 11 | NO |
| D08A0881 | Female | 76 | T3 | / | Alive | 77 | NO |
| D08A0882 | Male | 60 | T3 | Moderate | Dead | 8 | NO |
| D08A0883 | Male | 61 | T3 | Moderate | Alive | 77 | NO |
| D08A0912 | Male | 64 | T3 | Low | Dead | 31 | NO |
| D08A0913 | Male | 62 | T3 | Moderate | Dead | 28 | NO |
| D08A0950 | Male | 85 | T3 | High | Dead | 19 | NO |
| D08A0956 | Male | 60 | T3 | Moderate | Dead | 9 | NO |
| D08A0983 | Male | 73 | T2 | Low | Dead | 19 | NO |
| D08A1036 | Male | 69 | T2 | Low | Dead | 7 | NO |
| D08A1037 | Female | 75 | T2 | Low | Alive | 73 | NO |
| D08A1039 | Male | 84 | T3 | High | Dead | 5 | NO |
| D08A1087 | Male | 57 | T3 | Moderate | Alive | 72 | NO |
| D08A1095 | Male | 54 | T3 | Low | Dead | 12 | NO |
| D08A1123 | Female | 77 | T4b | Moderate | Dead | 2 | NO |
| D08A1223 | Male | 59 | T2 | High | Alive | 69 | NO |
| D08A1411 | Female | 78 | T3 | Moderate | Dead | 1 | NO |
| D08A1455 | Male | 62 | T3 | Low | Alive | Lost to follow-up | NO |
| D08A1458 | Male | 74 | T3 | High | Dead | 26 | NO |
| D08A1502 | Male | 56 | T3 | High | Dead | 13 | NO |
| D08A1505 | Male | 65 | T3 | High | Dead | 45 | NO |
| D08A1683 | Male | 60 | T3 | High | Dead | 3 | NO |
| D08A1910 | Female | 80 | T1b | Moderate | Alive | 58 | NO |
| D08A1964 | Male | 73 | T3 | High | Alive | 55 | NO |
| D08A1967 | Male | 60 | T3 | High | Alive | Lost to follow-up | NO |
| D08A1966 | Male | 64 | T3 | High | Dead | 1 | NO |
